# Discovery of Potent Dengue Virus NS2B-NS3 Protease Inhibitors Among Glycyrrhizic Acid Conjugates with Amino Acids and Dipeptides Esters

**DOI:** 10.3390/v16121926

**Published:** 2024-12-17

**Authors:** Yu-Feng Lin, Hsueh-Chou Lai, Chen-Sheng Lin, Ping-Yi Hung, Ju-Ying Kan, Shih-Wen Chiu, Chih-Hao Lu, Svetlana F. Petrova, Lidia Baltina, Cheng-Wen Lin

**Affiliations:** 1 Department of Medical Laboratory Science and Biotechnology, Asia University, Taichung 41354, Taiwan; yflin@asia.edu.tw; 2 Division of Hepato-Gastroenterology, Department of Internal Medicine, China Medical University Hospital, Taichung 404327, Taiwan; t674233@ms54.hinet.net; 3 School of Chinese Medicine, China Medical University, Taichung 404328, Taiwan; 4 Division of Gastroenterology, Kuang Tien General Hospital, Taichung 433401, Taiwan; b8401126@yahoo.com.tw; 5 Department of Medical Laboratory Science and Biotechnology, China Medical University, Taichung 404328, Taiwan; h840528hank@yahoo.com.tw (P.-Y.H.); s0975782938@gmail.com (J.-Y.K.); 6 The Ph.D. Program of Biotechnology and Biomedical Industry, China Medical University, Taichung 404328, Taiwan; 7 Institute of Bioinformatics and Systems Biology, National Yang Ming Chiao Tung University, Hsinchu 300093, Taiwan; wendy27chiu@gmail.com (S.-W.C.); chlu@nycu.edu.tw (C.-H.L.); 8 Ufa Institute of Chemistry, Ufa Federal Research Centre of RAS, Ufa 450054, Russia; petrova_sf89@anrb.ru

**Keywords:** glycyrrhizic acid, conjugates, amino acids, dipeptides, synthesis, antiviral activity, dengue virus type 2, NS2B-NS3 protease

## Abstract

This study investigated a library of known and novel glycyrrhizic acid (GL) conjugates with amino acids and dipeptide esters, as inhibitors of the DENV NS2B-NS3 protease. We utilized docking algorithms to evaluate the interactions of these GL derivatives with key residues (His51, Asp75, Ser135, and Gly153) within 10 Å of the DENV-2 NS2B-NS3 protease binding pocket (PDB ID: 2FOM). It was found that compounds **11** and **17** exhibited unique binding patterns, forming hydrogen bonds with Asp75, Tyr150, and Gly153. Based on the molecular docking data, conjugates **11** with L-glutamic acid dimethyl ester, **17** with β-alanine ethyl ester, and **19** with aminoethantic acid methyl ester were further demonstrated as potent inhibitors of DENV-2 NS3 protease, with IC50 values below 1 μM, using NS3-mediated cleavage assay. Compound **11** was the most potent, with EC50 values of 0.034 μM for infectivity, 0.042 μM for virus yield, and a selective index over 2000, aligning with its strong NS3 protease inhibition. Compound **17** exhibited better NS3 protease inhibition than compound **19** but showed weaker effects on infectivity and virus yield. While all compounds strongly inhibited viral infectivity post-entry, compound **19** also blocked viral entry. This study provided valuable insights into the interactions between active GL derivatives and DENV-2 NS2B-NS3 protease, offering a comprehensive framework for identifying lead compounds for further drug optimization and design as NS2B-NS3 protease inhibitors against DENV.

## 1. Introduction

The search and development of new antiviral agents for treating and preventing viral infections is critical in modern chemistry and virology. This urgency is driven by the widespread prevalence of dangerous viral infections such as HIV, hepatitis B and C, influenza A, avian and swine influenza, dengue fever, and Japanese encephalitis, as well as the emergence of new viral infections with pandemic potential like Ebola, Zika, Chikungunya, West Nile, and SARS-CoV-2 [1,2,3,4]. Pathogenic flaviviruses, including yellow fever, Japanese encephalitis, dengue, Zika, and West Nile viruses, are particularly concerning due to their mosquito-borne transmission. Aedes aegypti is a primary vector for several of these diseases [4,5,6]. Dengue virus (DENV) is one of the most geographically widespread flaviviruses, divided into four serotypes (DENV1-DENV4) [7,8,9]. It causes dengue fever, dengue hemorrhagic fever, and dengue shock syndrome. DENV2 alone is responsible for 50–100 million cases of dengue fever annually, potentially affecting 3 billion people, particularly in tropical and subtropical regions [7]. There is an increasing number of imported dengue fever cases in Europe, including Germany, the UK, and France. Currently, no antiviral drugs specifically target dengue infection, and existing antiviral treatments often have side effects and lead to drug resistance [10,11,12,13]. Hence, developing new antiviral agents against dengue infections is both urgent and complex.

DENV is an enveloped virus with icosahedral symmetry, characterized by a single-stranded RNA genome approximately 11 kb in length. This genome encodes three structural proteins—core (C), pre-membrane/membrane (prM/M), and envelope (E)—and seven non-structural proteins: NS1, NS2a, NS2b, NS3, NS4a, NS4b, and NS5, all within a single open reading frame [14,15,16]. Among these, the envelope (E) protein plays a critical role in host cell attachment and viral entry. The NS2B-NS3 protease facilitates the cleavage of the polyprotein at specific junctions, releasing individual non-structural proteins. Additionally, the NS5 protein, which possesses RNA helicase, methyltransferase (MTase), and RNA-dependent RNA polymerase (RdRp) activities, unwinds double-stranded RNA, modifies guanine to form the RNA genome’s cap structure, and synthesizes both positive- and negative-strand genomic RNAs [17]. These proteins are vital for viral replication and represent promising targets for the development of anti-DENV inhibitors. DENV NS2B/NS3 protease inhibitors, such as policresulen, strongly bind to its catalytic triad (His51, Asp75, and Ser135), demonstrating potential against multiple DENV serotypes and related viruses [18,19].

Plant triterpenoids have garnered significant attention as promising natural compounds for antiviral drug development [20,21,22,23]. Glycyrrhizic acid (GL), a main bioactive triterpene saponin from licorice roots (Glycyrrhiza glabra L. and Gl. uralensis Fisher), is particularly notable. Used in medicine for over 5000 years, GL exhibits a wide range of biological and pharmacological activities, including anti-inflammatory, antiulcer, antiallergic, hepatoprotective, antioxidant, antitumor, and antiviral properties [24,25,26,27,28,29,30,31]. GL inhibits several DNA and RNA viruses, including HIV, and is clinically used to treat chronic viral hepatitis [28,29,30,31]. It has also been found to inhibit the replication of coronaviruses SARS-CoV and SARS-CoV-2 [32,33]. GL and its derivatives can enhance the production of interferon-γ, making it suitable for stimulating nonspecific immunity and serving as a valuable preventive agent against various infections. The chemical modification of GL significantly impacts the antiviral properties of its derivatives and analogs, presenting a promising route for developing highly active antiviral compounds [29,30]. Semisynthetic derivatives of GL have proven to be highly effective inhibitors of SARS-CoV [34], HIV-1 [29], influenza A/H1N1 [35], and Epstein–Barr viruses [36]. Recently, GL conjugates with amino acids have shown promise as inhibitors of dengue and Zika viruses [37,38,39]. Conjugating natural compounds with amino acids is an attractive and effective strategy in drug discovery, leading to the development of pharmacologically active molecules [40].

This study explored a series of GL conjugates with amino acid and dipeptide esters as inhibitors of the DENV NS2B-NS3 protease using in silico prediction. Molecular docking identified key NS3 residues involved in hydrogen bonding with the active derivatives. The inhibitory effects were evaluated on DENV-2 NS2B-NS3 protease activity, viral infectivity, virus yield, and replication cycle. The results highlighted the antiviral efficacy and selectivity index of the GL derivatives, providing valuable insights into their potential as NS2B-NS3 protease inhibitors against DENV.

## 2. Materials and Methods

### 2.1. Chemistry

#### 2.1.1. General Information

All chemicals were used as commercially available. N-hydroxysuccinimide, N,N’-diciclohexylcarbodiimide, Reagent Woodword K, N-ethylmorpholine, and triethyl amine were purchased from ACROS organics and Sigma-Aldrich (St. Louis, MI, USA). Methyl and ethyl esters of amino acids as hydrochlorides, BocGlyONp, BocLeuONp, and BocIleONp, were purchased from Alfa Eaesar Co. (Haverhill, MA, USA) or Reanal (Budapest, Hungary). Methyl esters of γ-aminobutyric, ω-aminoenanthic, and 11-aminoundecanoic acids were produced by esterification of amino acids with MeOH in the presence of SOCl_2_ [41]. GL had a purity of 96% according to HPLC [38]. The standard procedures purified the solvents.

Brüker AVANCE-III pulse spectrometer (Bruker Optik GmbH, Ettlingeh, Germany) (^1^H 500 MHz and ^13^C 125 MHz) was used for the measurement of NMR spectra in CD_3_OD or DMSO-d_6_. Tetramethylsilane was the internal standard. Chemical shifts are detected in δ (ppm), the coupling constants (*J*)-in Hertz (Hz). The registration of IR spectra was made on Prestige-21 spectrophotometer (Shimadzu; Kyoto, Japan). Optical activity was determined using the Perkin-Elmer 341 MC polarimeter (PerkinElmer, Waltham, MA, USA) with a sodium lamp (589 nm). HPLC analysis performed on Shimadzu LC-20 liquid chromatograph equipped with a UV detector at 254 nm, using reversed-phase columns such as Vydac 218TP C18 (250 × 4.6 mm; 5 μM) (SUPELCO, Saint-Louis, MI, USA), Pursuit C18 (250 × 4.6 mm; 5 μM) (Agilent Technologies, Santa Clara, CA, USA); Atlantis C18 (250 × 4.6 mm; 5 μM) (Waters, Milford, CT, USA); Hypersil ODS C18 (250 × 4.6 mm; 5 μM) (MZ-Analysentechnik GmbH, Mainz, Germany); Zorbax RX C18 (250 × 4.6 mm; 5 μM) (Agilent Technoologies, Santa Clara, USA); Kromasil C18 (250 × 4.6 mm; 5 μM) (MZ-Analysentechnik GmbH, Mainz, Germany). Methanol was used as a mobile phase with a flow rate of 1 mL/min. HPLC data for GL derivatives are given in Supplementary Figures S1–S25; NMR spectra for novel compounds are in Supplemental Figures S26–S41.

The purification of all compounds was carried out by column chromatography (CC) on silica gel (Si gel) KSK SG (50–150 mesh fraction, Sorbpolimer) with TLC control, which was performed on the Sorbfil plates (Sorbpolymer, Krasnodar, Russia) using benzene–ethanol or chloroform–ethanol mixtures (5:1, v%). Spots were detected by the solution of 20% phosphotungstic acid in ethanol with subsequent heating at 200–210 °C for 2–3 min.

#### 2.1.2. A General Procedure to Synthesize Compounds **2**–**8**

Glycyrrhizic acid (0.82 g, 1 mmol) was dissolved in DMF (30 mL) or THF-DMF (1:1), then N-hydroxybenzotriazol (3.5 mmol) and N,N′-dicyclohexylcarbodiimide (3.5 mmol) were added to a mixture under stirring at room temperature (RT) (20–22 °C). The reaction mixture was stirred for 5–6 h, the formed precipitate of N, N′-dicyclohexylurea was filtered off, and amino acids esters hydrochlorides (3.5–4.0 mmol) were added to the solution; then, the excess (9.0–10.0 mmol) of trimethylamine was added. After keeping the reaction mixture for 22–24 h with a periodic stirring, it was diluted with ice water (100 mL) and acidified with citric or 5% hydrochloric acids until pH 3–4. The resulting precipitate was separated and dried. The target compounds were isolated by CC on Si gel using CHCl_3_-MeOH-H_2_O mixtures (400:10:1, 200:10:1, 100:10:1, 50:10:1, *v*/*v*) with TLC control; HPLC analyzed the isolated GL derivatives. NMR data of compounds **2**–**5** corresponded to the literature ones [42], and **3** [43]. The IR, ^1^H NMR, and ^13^C NMR data for the compounds **2**–**8** are provided in the Supplementary Materials.

#### 2.1.3. A General Procedure to Synthesize Compounds **9**–**20**

Glycyrrhizic acid (0.82 g, 1 mmol) was dissolved in tetrahydrofurane (30 mL) and the solution was cooled in the ice bath to 0–5 °C; then, N-hydroxysuccinimide (0.6 g, 5.2 mmol) and N, N′-dicyclohexylcarbodiimide (0.5–0.6 g, 2.5–3.0 mmol) were added, and the reaction mixture was stirred at 0–5 °C for 1 h, then at 20–22 °C for 5 h. The resulting precipitate of N, N′-dicyclohexylurea was separated, and to the solution, amino acid ester hydrochlorides (2.5–3.0 mmol) were added, followed by the excess of triethylamine (7.0–8.0 mmol). After keeping the mixture with periodic stirring at 20–22 °C for 22–24 h, it was diluted with ice water and acidified with citric or 5% hydrochloric acids to pH 3–4. The precipitated product was filtered off, dried, and purified by CC as above. The purity of all compounds was analyzed by HPLC. NMR data of compounds **9**–**13**, **16**, **17,** and **20** agree with the literature ones: **9**, **10**, **13**, **16**, **17** [38], **11** [44,45], **12** [44], **18**, and **20** [41]. The IR, ^1^H NMR, and ^13^C NMR data for the compounds **14**, **15**, and **19** are provided in the Supplementary Materials.

#### 2.1.4. 3-O-{2-O-[N-(β-D-glucopyranosyluronoyl)-glycyl-L-tyrosine methyl ester]-N-(β-D-glucopyranosyluronoyl)-glycyl-L-tyrosine methyl ester}-(3β,20β)-11-oxo-30-noroleane-12-ene-30-Acid **21**

A. *Synthesis of dipeptide BocGly-TyrOMe.* To a solution of 4-nitrophenyl ester of tert-butyloxycarbonyl glycine (BocGlyONp) (1.48 g, 5 mmol) in DMF (20 mL) N-ethylmorpholine (0.7 mL, 6 mmol) and L-tyrosine methyl ester hydrochloride (1.16 g, 5 mmol) were added, and a reaction mixture was stirred at 45–50 °C for 48 h. Then it was evaporated to half the volume, dissolved in ethylacetate, and washed with a 5% water solution of NaHCO_3_ (until the disappearance of the yellow color), water, 5% hydrochloric acid, and water again. An organic phase was dried over MgSO_4_ and evaporated. A residue was re-precipitated from ethylacetate–hexane and was homogenous by TLC and HPLC analysis: R_f_ = 0.57 (benzene–ethanol, 10:1); HPLC (99.2%, τ 3.08 min, Discovery) (Supplemental Figure S20). The yield was 1.50 g (85%). IR (ν, sm^−1^): 3450-3200 (NH), 1735 (COOMe), 1668 (C=O), 1615 (Tyr), 1593 (CONH), 1516 (Tyr), 1500 (Tyr). Anal. calcd. for C_17_H_24_N_2_O_6_ C 57.44, H 6.86, N 7.95%; found, C 57.25; H 6.76; N 7.80%. It was used for synthesis without a further purification.

B. Dipeptide BocGly-TyrOMe (1.5 g) was dissolved in CF_3_COOH (10 mL) and stirred for 40 min at 20–22 °C, then evaporated and worked up with diethyl ether to receive CF_3_COOH • Gly-TyrOMe, which was used in a reaction with GL.

C. *Synthesis of conjugate **21***. To a solution of GL (0.82 g, 1 mmol) in DMF (20 mL), N-ethylmorpholine (0.7 mL, 6 mmol) and a Woodward’s reagent K (0.63 g, 2.5 mmol) were added at 0–5 °C. A mixture was stirred at this temperature for 1.5 h and at 20–22 °C for 1.5 h. Then, N-ethylmorpholine (0.5 mL) and CF_3_COOH • Gly-TyrOMe (0.92 g, 2.5 mmol) were added and a reaction mixture was kept at 20–22 °C for 48 h with periodic stirring. Then, it was evaporated to dryness and a residue was purified on a silica gel column using a gradient mixture CHCl_3_-MeOH-H_2_O (400:10:1→50:10:1, v%) with TLC control. Compound **21** was isolated as an amorphous solid, yielding 55%. HPLC (98.5%, τ 2.42 min, Zorbax) (Supplemental Figure S21). The IR, ^1^H NMR, and ^13^C NMR data for compound **21** are provided in the Supplementary Materials.

#### 2.1.5. 3-O-{2-O-[N-(β-D-glucopyranosyluronoyl)-L-isoleucine-tyrosine methyl ester)]-N-(β-D-glucopyranosyluronoyl)-L-isoleucine-tyrosine methyl ester)}-(3β,20β)-11-oxo-30-noroleane-12-ene-30-oic Acid **22**

A. *Synthesis of Boc Ile-TyrOMe*. To a solution of 4-nitrophenyl ester of tert-butyloxycarbonyl L-isoleucine (BocLeuONp) (1.76 g, 5 mmol) in DMF (20 mL), N-ethylmorpholine (0.7 mL) and L-tyrosine methyl ester hydrochloride (1.15 g, 5 mmol) were added and a reaction mixture was kept at 45–50 °C for 48 h with a periodic stirring. Then, it was evaporated to half the volume, dissolved in CHCl_3_, and washed with 5% NaHCO_3_ water solution (until the disappearance of the yellow color), water, 5% hydrochloric acid, and water again. An organic phase was dried over MgSO_4_ and evaporated. A residue was re-precipitated from ethylacetate–hexane and was homogenous by TLC and HPLC (97.3%, τ 3.10 min; the Discovery column) (Supplemental Figure S22); R_f_ = 0.57 (benzene–ethanol, 5:1). IR (ν, sm^−1^): 3400–2800 (CONH), 1745 (COOMe), 1660 (C=O), 1615 (Tyr), 1593 (CONH), 1515 (Tyr). Yield was 1.67 g (84%). Anal. calc. for C_21_H_22_N_4_O_6_ C 63.21, H 5.55, N 7.02%; found, C 63.10, H 5.43, N 6.85%. M = 398.98. It was used for synthesis without a further purification.

B. Dipeptide BocIle-TyrOMe (1.67 g) was dissolved in CF_3_COOH (10 mL) and stirred at 20–22 °C for 40 min, evaporated, and worked up with diethyl ether to produce CF_3_COOH • Leu-TyrOMe, which was used in a reaction with GL.

C. *Synthesis of GL conjugate **22**.* To a solution of GL (0.82 g, 1 mmol) in DMF (20 mL), N-ethylmorpholine (0.7 mL, 6 mmol) and Woodward’s reagent K (0.6 g, 2.5 mmol) were added at 0–5 °C and stirred at 0–5 °C for 1.5 h and at 20–22 °C for 1.5 h. Then, N-ethylmorpholone (0.7 mL) and CF_3_COOH•Ile-TyrOMe (1.12 g, 3 mmol) were added, and a reaction was kept at 20–22 °C for 48 h with a periodic stirring. Then, it was evaporated, and the residue was subjected to column chromatography, as described for **21**. The compound **22** was isolated as an amorphous solid, yielding 54%; [α]_D_^20^ + 49° (*c* 0.04, EtOH). IR (ν, sm^−1^): 3500-3200 (OH, NH), 1730 (COOMe), 1661 (C=O), 1652 (C=O), 1592 (CONH), 1560 (CONH), 1519, 1500 (Tyr). HPLC (96.0%, τ 2.56 min, the Vydac) (Supplemental Figure S23). The ^1^H NMR and ^13^C NMR data for compound **22** are provided in the Supplementary Materials.

#### 2.1.6. 3-O-{2-O-[N-(β-D-glucopyranosyluronoyl)-L-isoleucine-phenylanine methyl ester)]-N-(β-D-glucopyranosyluronoyl)-L-isoleucine-phenylalanine methyl ester)}-(3β,20β)-11-oxo-30-noroleane-12-ene-30-oic Acid **23**

A. *Synthesis of dipeptide BocIle-PheOMe.* To a solution of 4-nitrophenyl ester of tert-butyloxycarbonyl isoleucine (BocIleONp) (1.76 g, 5 mmol) in DMF (20 mL), N-ethylmorpholine (0.7 mL, 6 mmol) and L-phenylalanine methyl ester hydrochloride (1.0 g, 5 mmol) were added, and a reaction mixture was stirred at 45–50 °C for 48 h. Then it was evaporated to half the volume, dissolved in ethylacetate, and washed with a 5% water solution of NaHCO_3_ (until the disappearance of the yellow color), water, 5% hydrochloric acid, and water again. An organic phase was dried over MgSO_4_ and evaporated. A residue was re-precipitated from ethylacetate–hexane and was homogenous by TLC and HPLC analysis (99.8%, τ 3.08, Discovery) (Supplemental Figure S24); R_f_ = 0.54 (benzene–ethanol, 5: 1). IR (ν, sm^−1^): 3450-2900 (BocNH, CONH), 1744 (COOMe), 1656 (C=O), 1617 (Ph), 1593 (CONH), 1520, 1501 (Phe). Yield was 1.30 g (82%). Anal. calcd. for C_20_H_31_N_4_O_5_, C 76.02, H 9.87, N 8.87%; found, C 75.82, H 9.73, N 8.75%. M = 315.96. It was used for synthesis without a further purification.

B. Dipeptide BocIle-PheOMe (1.30 g) was dissolved in CF_3_COOH (10 mL) and stirred for 40 min at 20–22 °C, then evaporated and worked up with diethyl ether to receive CF_3_COOH • Ile-PheOMe, which was used in a reaction with GL.

C. *Synthesis of conjugate **23**.* To a solution of GL (0.82 g, 1 mmol) in DMF (20 mL), N-ethylmorpholine (0.7 mL, 6 mmol) and a Woodward’s reagent K (0.63 g, 2.5 mmol) were added at 0–5 °C. A mixture was stirred at this temperature for 1.5 h and at 20–22 °C for 1.5 h. Then, N-ethylmorpholine (0.5 mL) and CF_3_COOH • Ile-PheOMe (1.0 g, 2.5 mmol) were added, and a reaction mixture was kept at 20–22 °C for 48 h with a periodic stirring. Then, it was evaporated to dryness, and a residue was purified on a silica gel column, as described for **21** with TLC control.

### 2.2. Hierarchical Clustering and Molecular Docking for the Complexes of the Compounds with the Catalytic Dyad of DENV-2 NS3 Protease

The functional significance of atoms and substructures in antiviral actions was examined through a comprehensive analysis of three-dimensional interactions between DENV-2 NS2B-NS3 protease and GL derivatives using iGEMDOCK [46], and PyMOL drew the interactions. The catalytically active form of DENV-2 NS2B-NS3 protease (PDB ID: 2FOM [47]) with the lowest resolution was selected as the target protein for virtual screening. The binding site in the DENV-2 NS2B/NS3 protease was defined by residues within a 10Å radius of His51, Asp75, Ser135, or Gly153. To evaluate the potential binding of 24 compounds to the active sites of NS2B/NS3 protease, iGEMDOCK, known for its accurate predictions, was employed, following the methodology from a previous study [48]. The docking process analyzed three types of interactions: electrostatic forces (E), hydrogen bonding (H), and van der Waals forces (V). A hierarchical tree illustrated compound similarities, with interaction codes indicating whether the forces occurred in the main chain (M) or side chain (S) of DENV-2 NS3 protease. These 24 compounds were then clustered based on the types of interactions between their moieties and the protein residues.

### 2.3. Antiviral Experiments

#### 2.3.1. Trans-Cleavage Assays with DENV-2 NS2B-NS3 Protease

In our previous study [49], a trans-cleavage assay for DENV-2 NS2B-NS3 protease was established using cells expressing CFP-trrg-YFP, which were transiently transfected with pcDNA-DEN2_NS2BNS3. These transfected cells were treated with indicated compounds at various concentrations (0.001, 0.01, 0.1, 1, and 10 μM). After 24 h of treatment, FRET signals were measured using the SpectraMax Multi-Mode Microplate Reader at 436–528 nm emission wavelengths. The difference in FRET intensity between CFP-trrg-YFP-expressing cells and those transiently transfected with pcDNA-DEN2_NS2BNS3 was set as 100% DENV-2 NS2B-NS3 protease activity. The relative residual enzymatic activity in treated cells was compared to untreated controls and used to create inhibition curves for each compound. The half-maximal inhibitory concentration (IC50) was determined from these inhibition curves to assess the compounds’ effects on DENV-2 NS2B-NS3 protease activity.

#### 2.3.2. Cytopathic Effect (CPE), In Vitro Infectivity, and Virus Yield Assays with DENV-2

Vero E6 cells, originating from the kidney epithelium of African green monkeys, were maintained in DMEM enriched with 10% fetal bovine serum (FBS) and penicillin/streptomycin. These cells were used to propagate viral stocks of the DENV-2 strain 16681 [37,38,49] and to assess the antiviral effects of GL derivatives. Cytopathic effect (CPE) and in vitro infectivity assays were performed using Vero E6 cells. For both assays, a fixed quantity of DENV-2 at a multiplicity of infection (MOI) of 0.05 was introduced into each well, and cells were treated with compounds 11, 17, and 19 at varying concentrations (0.001, 0.01, 0.1, 1, and 10 μM). After 96 h of incubation, CPEs in DENV-2-infected cells were captured using microscopy. The in vitro infectivity of DENV-2 was assessed by calculating the ratio of NS4B-positive cells to the total number of DAPI-stained cells, utilizing an immunofluorescent staining assay with anti-DENV NS4B antibodies (GeneTex, Inc., Taiwan). For the virus yield assay, 10-fold serial dilutions of cultured media from DENV-2-infected cells were added to Vero E6 cells in 96-well plates with or without GL derivatives. After 96 h of incubation, the number of CPEs in each well was counted to calculate the mean tissue culture infectious dose (TCID50) per milliliter, representing virus yield. Virus yield from mock-treated/infected cells was set as the reference (100%), and the relative residual virus yield in treated/infected cells was determined. The half-maximal effective concentration (EC50) for viral infectivity or yield was calculated using a linear regression calculator. In addition, Vero E6 cells were treated with the compounds **11**, **17**, and **19** (0, 0.1, 10, 50, and 100 μM) for cytotoxicity assessment. After 96 h at 37 °C, MTT solution was added for 4 h, followed by DMSO for 15 min. Absorbance at 570 nm quantified viability, and the 50% cytotoxic concentration (CC50) was calculated from the concentration–response curve. The selectivity index (SI) was the ratio of EC50 to IC50.

#### 2.3.3. Time-of-Addition/Removal Assays with DENV-2

Time-of-addition/removal assays were conducted to evaluate the inhibitory effects of compounds **11**, **17**, and **19** at different stages of DENV-2 replication, including the NS2B-NS3-mediated phase (post-entry), the cellular antiviral state, viral entry stages [38,47,48]. In the pre-treatment mode, cells in 6-well plates were treated with the compounds for 2 h at 37 °C, washed with PBS, incubated with DENV-2 for 2 h, and washed again. In the co-treatment mode, cells were simultaneously incubated with DENV-2 and the compounds for 2 h at 37 °C, followed by a PBS wash. For the post-infection treatment, cells were first infected with DENV-2 for 2 h, then treated with the compounds for 2 h at 37 °C, followed by a PBS wash. After an 18 h incubation in all modes, the treated or untreated DENV-2-infected cells were analyzed using immunofluorescent staining to assess residual infectivity. This was determined by calculating the ratio of NS4B-positive cells to the total number of DAPI-stained cells. The compounds’ inhibitory effects on various stages of DENV-2 replication were evaluated based on the level of residual infectivity.

#### 2.3.4. Statistical Analysis

Data were analyzed with One-way ANOVA and Scheffe’s post hoc test using SPSS 12.0. Statistical significance set was at *p* < 0.05.

## 3. Results

### 3.1. Synthesis of GL Conjugates

**Figure 1:**
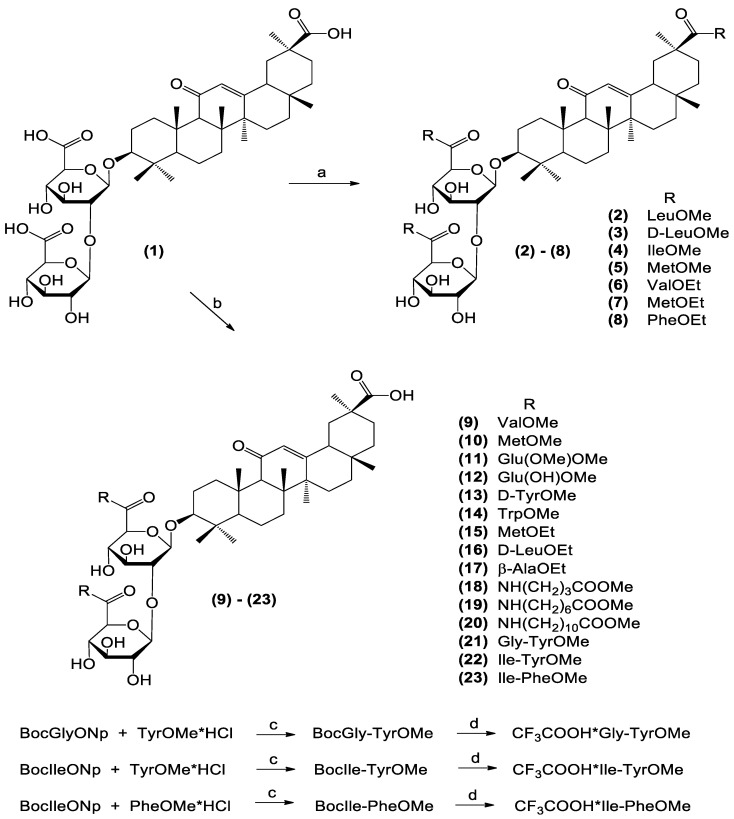
Synthesis of GL conjugates. Reagents and conditions: (**a**) HOBt/DCC, 5–6 h, RT; AK, Et_3_N, DMF or THF-DMF, 24 h, RT; (**b**) HOSu/DCC, 5–6 h, 0–5 °C; AK, Et_3_N, THF, 24 h, RT; (**c**) NEM, DMF, 45–50 °C, 48 h; (**d**) 1. CF_3_COOH, 40 min, RT; 2. DMF, NEM, RWK, 0–5 °C, 1.5 h, RT 1.5 h; 3. CF_3_COOH•dipeptide ester, NEM, RT, 48 h.

A library of 23 known and novel GL conjugates with three or two alkyls (methyl/ethyl) esters of L- and D-amino acids, including long-chaired amino acids and dipeptides, were synthesized as shown in Figure 1. GL conjugates **2**–**8** containing amino acid residues in the carbohydrate and triterpene parts were produced by using N-hydroxybenzotriazol (HOBt) and N, N′-dicyclohexylcarbodiimide (DCC) for the activation of COOH groups with yields 75–85%. Compounds **2**–**5** have been previously described [42,43] and were first characterized by HPLC (Supplemental Figures S1–S4) and ^1^H NMR (500 MHz) spectra (see Supplementary Materials).

The second group of GL conjugates were compounds **9**–**21** bearing amino acids alkyl (methyl/ethyl) esters only in the carbohydrate part of glycoside, including long-chaired amino acids. They have been synthesized by using the HOSu-DCC method [38,41,44] with yields of 55–58%. Compounds **14**, **15**, and **19** are novel GL derivatives. The NMR spectral data and HPLC analysis for compounds **9**, **10**, **13**, **16**, and **17** corresponded to those published in [38], for compounds **11** and **12** in [44,45], and for compounds **18** and **20** in [41].

The third group of GL derivatives were conjugates with dipeptides **21**–**23** containing methyl esters of aromatic amino acids (Tyr, Phe) as the second amino acid. These compounds were synthesized as shown in Figure 1. Firstly, tert-butyloxycarbonyl (Boc) protected dipeptides were produced by the activated esters method from Boc-amino acids 4-nitrophenyl (Np) esters (Boc-GlyONp, Boc-LeuONp, Boc-IleONp) and methyl esters of tyrosine and phenylalanine hydrochlorides in DMF in the presence of N-ethylmorpholine (NEM) at 45–50 °C with yields 80–85%. Boc-protected dipeptides were treated with CF_3_COOH to delete the Boc groups and used in the coupling reaction with GL as CF_3_COOH salts. Conjugation with GL was carried out with Reagent Woodword K (RWK) in DMF in the presence of an excess of NEM. The target dipeptide conjugates **21**–**23** were isolated by column chromatography with 52–55% yields. All compounds produced had a purity of more than 95% according to the HPLC data (Supplemental Figures S1–S25). The structures of the novel compounds **6**–**8**, **14**, **15**, **19**, and **21**–**23** were confirmed by IR and NMR (^1^H-500 MHz and ^13^C-125 MHz) spectra and elemental analysis. The NMR spectra for the novel GL derivatives are given in Supplementary Materials (Supplemental Figures S26–S45).

### 3.2. Computational Analysis of the Interaction Between GL Derivatives and DENV NS2B-NS3 Proteases

**Figure 2:**
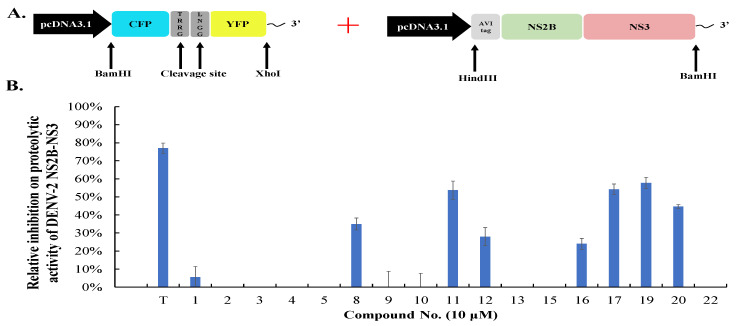

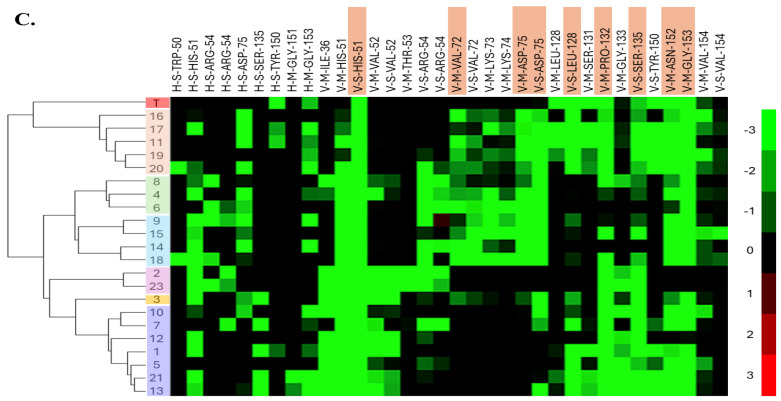
Hierarchical clustering and interaction analysis of GL (compound **1**) and its derivatives (compounds **2–23**) with the enzymatic site of DENV-2 NS3 protease, along with their inhibitory assay results, were performed. A quantitative FRET-based cleavage assay was used to evaluate inhibitory effects. Cells expressing the FRET substrate CFP-trrg-YFP (panel **A**, **left**) were transiently transfected with pcDNA3.1_DENV-2 NS2B-NS3 (panel **B**, **right**) and treated with representative compounds at 10 μM for 48 h. FRET signaling (436–528 nm) was measured, with intensity in mock-transfected cells set as 100%. Residual inhibition in compound-treated cells was compared to untreated controls (panel **B**). A hierarchical clustering tree generated by iGEMDOCK displayed the interaction profile of the GL derivatives (panel **C**). Compounds are listed along the y-axis, while interactive residues appear on the x-axis. Residues interacting with all Group I compounds (**11**, **16**, **17**, **19**, **20**) are marked in orange. Interaction codes indicate the type of force (hydrogen bond [H] or van der Waals [V]), the location of interaction (main chain [M] or side chain [S]), and the residue type with its position in DENV-2 NS3 protease.

**Figure 3:**
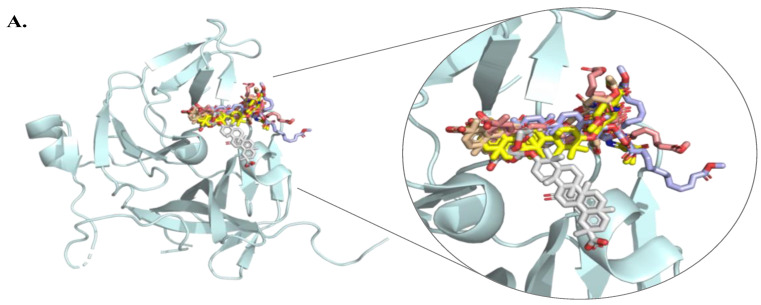

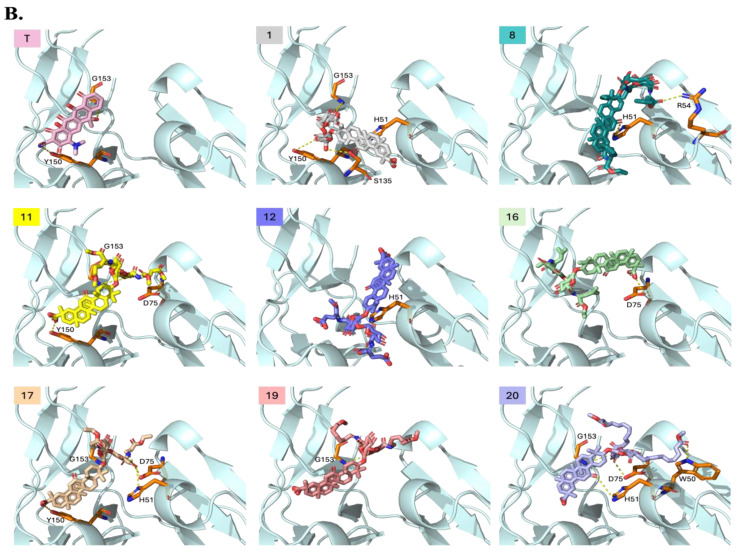
Interactions of molecular docking of tetracycline (compound T) and the compounds **1**, **8**, **11**, **12**, **16**, **17**, **19**, and **20** into the active site of DENV-2 NS3 protease analyzed by iGEMDOCK, and drew by PyMOL. The structures are presented in a zoomed-out view (**A**) and a zoomed-in view (**B**), depicted as cartoon representations, with interactive residues highlighted as orange sticks. The docked compounds are shown in different colors in a zoomed-out view (**A**) and a zoomed-in view (**B**). Yellow dotted lines indicate the hydrogen bonds.

The FRET substrate CFP-trrg-YFP was used to screen the inhibitory effects of various compounds on DENV-2 NS2B-NS3 protease activity (Figure 2A,B). Cells were transfected with pcDNA-CFP-trrg-YFP and pcDNA-DENV2_NS2BNS3, then treated with indicated compounds, including tetracycline (T, 10 μM) as a positive control [50], and a mock treatment (M). Compounds T, 8, 11, 12, 16, 17, 19, and 20 significantly reduced protease activity by more than 20% (Figure 2B). Subsequently, GL derivatives were evaluated as potential DENV-2 NS2B-NS3 protease inhibitors using docking algorithms, based on their ability to inhibit protease activity. These algorithms assessed interactions with residues within 10 Å of the catalytic triad—His51, Asp75, and Ser135—located in the binding pocket of the protease (PDB ID: 2FOM). Sequence comparisons confirmed that the catalytic activity depends on this triad, as substituting Ser135 with alanine rendered the enzyme inactive [47]. Previous studies on N-methylcytisine thio (mCy thio) derivatives revealed that mCy thio 6 formed hydrogen bonds with Gly153 and interacted via van der Waals forces with His51, Asp75, and Gly153 [49]. In the current study, GL (compound 1), its 22 derivatives, and tetracycline (compound T) were docked into the active site of the protease using iGEMDOCK 2.1 software. These compounds were clustered based on electrostatic, hydrogen-bonding, and van der Waals interactions to illustrate their binding modes (Figure 2C). Hierarchical clustering and interaction analysis grouped GL and its derivatives into five clusters (Groups I–V) based on their interactions with the DENV-2 NS2B-NS3 protease, using tetracycline’s binding profile as a reference. Group I, which included compounds **11**, **16**, **17**, **19**, and **20**, formed a distinct cluster because of specific interactions with key binding pocket residues His51, Asp75, Ser135, and Gly153 (Figure 3A,B). Among these, compounds **11** and **17** exhibited unique binding characteristics, forming hydrogen bonds with Asp75, Tyr150, and Gly153, and van der Waals forces with His51, Val72, Asp75, Leu128, Ser131, Pro132, Gly133, Ser135, Tyr150, Asn152, and Gly153. In contrast, compounds 16, 19, and 20 lacked hydrogen bonds with Tyr150, and der Waals forces with Gly133 (Figure 2C and Figure 3B). The inhibitory effects of these compounds on DENV-2 protease activity were further analyzed using a trans-cleavage assay at various concentrations to elucidate their structure–function relationships.

### 3.3. Inhibitory Effects of Compounds 11, 16, 17, 19, and 20 on DENV-2 NS3 Protease-Mediated Proteolysis

**Figure 4:**
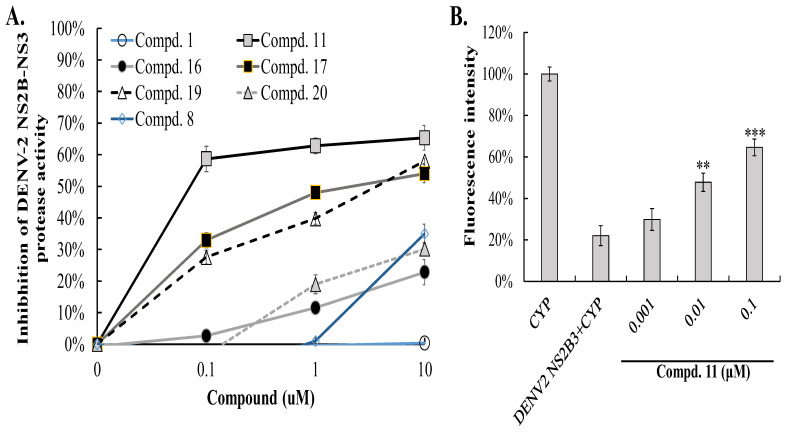
The inhibitory effects of GL and the compounds in Group I on the proteolytic activity of DENV-2 NS2B-NS3 protease were assessed using a quantitative trans-provided cleavage FRET assay. Cells expressing the FRET substrate CFP-trrg-YFP were transfected with pcDNA3.1_DENV-2 NS2B-NS3 and treated with compounds **1** (GL), **8**, **11**, **16**, **17**, **19**, and **20** at varying concentrations for 48 h. FRET signaling (436–528 nm) was measured, with mock-transfected cells set as 100% intensity. Residual enzymatic activity was compared to untreated controls to assess inhibition (**A**), highlighting FRET signals in compound 11-treated cells (**B**). ** *p* < 0.01; *** *p* < 0.001 compared to untreated cells expressing DENV2 NS2B3 and CYP.

The quantitative trans-provided cleavage FRET assay was used to assess the inhibitory effects of Group I compounds compared to GL (compound **1**). Compounds **11**, **17**, and **19** demonstrated dose-dependent increases in FRET signals from CFP-trrg-YFP, indicating significantly stronger inhibition of DENV-2 NS2B-NS3 protease activity. In contrast, compounds **16** and **20** showed weaker activity, with IC50 values exceeding 10 μM, while compound **1** exhibited no inhibitory effect (Figure 4A). Among the tested compounds, compound **11** displayed the most potent inhibition, with an IC50 of 0.0134 ± 0.002 μM, followed by compound **17** (IC50 = 0.34 ± 0.002 μM) and compound **19** (IC50 = 0.52 ± 0.02 μM) (Figure 4A,B, Table 1). These findings align with molecular docking predictions, suggesting that GL conjugates **11**, **17**, and **19** are promising candidates for further studies.

### 3.4. Antiviral Activity of Compounds 11, 17, and 19 Against DENV-2

**Figure 5:**
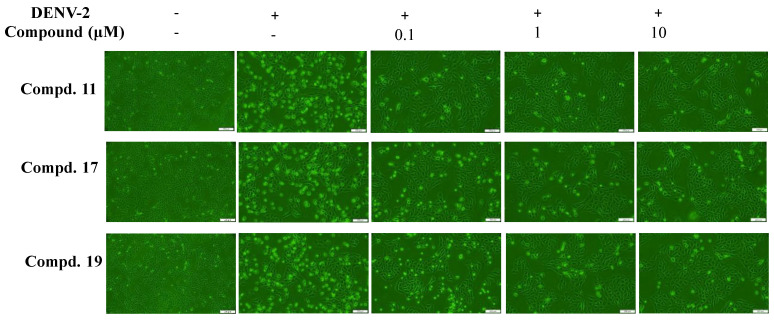
Inhibition of cytopathic effects induced by DENV-2 through compounds **11**, **17**, and **19**. Vero E6 cells were infected with DENV-2 at a MOI of 0.01 and treated immediately with varying concentrations of the indicated compounds. Light microscopy images were taken 96 h post-infection to capture the DENV-2-induced cytopathic effects. Scale bar = 200 µm.

**Figure 6:**
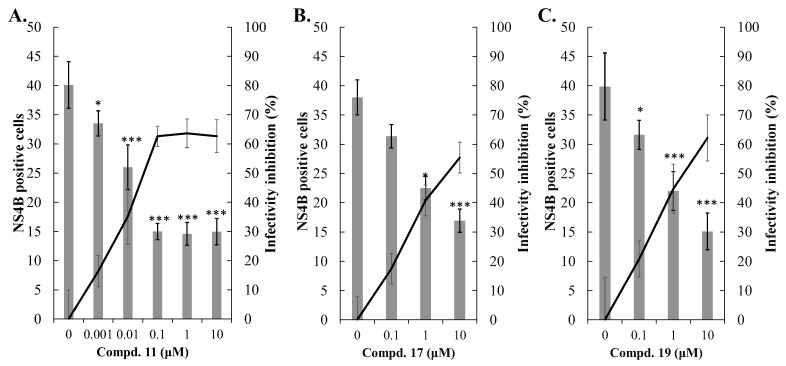
Inhibition of DENV-2 infectivity in vitro by active compounds. Vero E6 cells were infected with DENV-2 in the presence or absence of Compounds **11** (**A**), **17** (**B**), and **19** (**C**) at the indicated concentrations. After 96 h of incubation, the treated and infected cells were analyzed using immunofluorescence staining with anti-DENV-2 NS4B antibodies. The relative infectivity was calculated by the ratio of DENV-2 NS4B-positive cells to the total number of DAPI-stained cells. Relative inhibition activity was calculated by subtracting the residual infectivity from the full infectivity observed in untreated infected cells. * *p* < 0.05; *** *p* < 0.001 compared to untreated infected cells.

**Figure 7:**
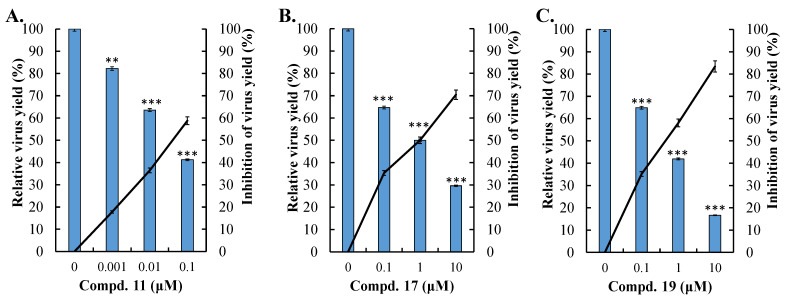
Reduction in DENV-2 yield in vitro by active compounds. Virus yield in the culture media of cells infected with DENV-2, with or without treatment by Compounds **11** (**A**), **17** (**B**), and **19** (**C**) at the indicated concentrations, was analyzed 96 h post-treatment using the TCID50 assay. Relative virus yield inhibition was determined by subtracting the residual yield in treated/infected cells from the full yield detected in untreated infected cells. ** *p* < 0.01; *** *p* < 0.001 compared to untreated infected cells.

Compounds **11**, **17**, and **19** were tested for their inhibitory effects on DENV-2-induced cytopathic effects (CPE), in vitro infectivity, and virus yield at concentrations of 0.1, 1, and 10 μM (Figure 5, Figure 6 and Figure 7, Table 1). All three compounds significantly reduced CPE levels, decreased the number of NS4B-positive cells, and suppressed virus yields in a concentration-dependent manner (Figure 5, Figure 6 and Figure 7). The CC50 values for all three compounds in Vero E6 cells exceeded 100 μM by the MTT assay (Figure S42). Among them, compound **11** showed the most potent antiviral activity, with EC50 values of 0.034 μM for in vitro infectivity and 0.042 μM for virus yield (Figure 6A and Figure 7A). Compound **17** exhibited antiviral activity with EC50 values of 4.34 μM for in vitro infectivity and 0.79 μM for virus yield (Figure 6B and Figure 7B), while compound **19** displayed EC50 values of 2.34 μM for in vitro infectivity and 0.43 μM for virus yield (Figure 6C and Figure 7C). The selective indices were over 2000 for compound **11**, **20** for compound **17**, and 40 for compound **19**. These results demonstrate that compound **11** possesses the strongest antiviral activity against DENV-2 among these three active compounds, consistent with their inhibitory effects on DENV-2 NS3 protease activity. Compound **17**, despite having the second-highest potency in inhibiting DENV-2 NS3 protease activity, showed weaker effects on in vitro infectivity and virus yield than compound **19**, which ranked third in potency for inhibiting the same protease. Thus, additional experiments were needed to explore the antiviral mechanisms of these three active compounds.

### 3.5. Anti-DENV-2 Action of Compounds **11**, **17**, and **19**

**Figure 8:**
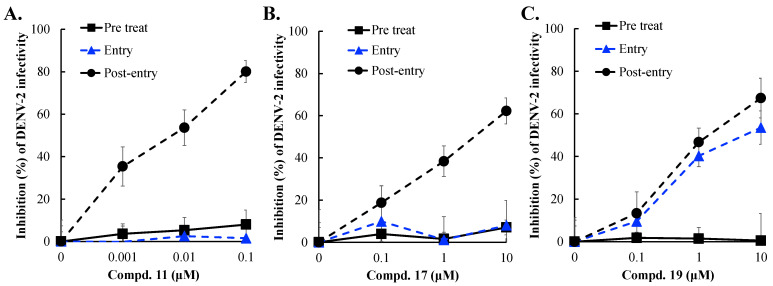
Time-of-addition and removal assay for the antiviral action of Compounds **11** (**A**), **17** (**B**), and **19** (**C**). In pre-treatment, cells were treated with the compounds for 2 h, washed, and then infected with DENV-2 for 2 h. In co-treatment, cells were infected with DENV-2 and treated simultaneously. In post-infection, cells were first infected and then treated for 2 h. After an 18 h incubation, residual infectivity was assessed by immunofluorescence and DAPI staining. DENV-2 inhibition was calculated as 1—(NS4B-positive cells in treated/NS4B-positive cells in controls).

To investigate whether compounds **11**, **17**, and **19** target the NS3 protease-mediated stage of DENV-2 replication, a time-of-addition/removal assay was performed in three distinct modes (Figure 8). In the first mode, pretreatment, cells were treated with the compounds before viral infection to assess their effect on the cellular antiviral state. In the second mode, treatment during entry, the compounds were added during the first two hours of infection to target the virus entry stage. In the third mode, treatment post-entry, the compounds were applied two hours after infection to evaluate their effect on the post-entry stage of the viral life cycle. None of the three compounds inhibited viral infectivity during the pretreatment mode at concentrations ranging from 0.001 to 10 μM. However, during the treatment at the entry stage, only compound **19** showed significant inhibition of viral infectivity, with an EC50 value of 5.03 μM, highlighting its effectiveness in blocking viral entry. In the post-entry treatment mode, all three compounds demonstrated strong inhibitory effects on viral infectivity, indicating their action on the NS3 protease-mediated replication stage. The EC50 values were 0.005 μM for compound **11**, 2.99 μM for compound **17**, and 1.91 μM for compound **19**. These findings highlight the superior anti-DENV activity of compounds **11** and **17**, which likely inhibit NS3 protease activity to block DENV-2 replication. In contrast, compound **19** exhibited dual antiviral actions, inhibiting the entry and post-entry stages of the viral life cycle.

## 4. Discussion

This study identified compounds **11**, **17**, and **19** as potent inhibitors of DENV-2 NS3 protease, with IC50 values below 1 μM, using a combination of in silico docking and NS3-mediated cleavage FRET assays (Figure 1, Figure 2, Figure 3 and Figure 4). These compounds are GL conjugates with amino acid alkyl esters exclusively in the carbohydrate part, such as L-glutamic acid dimethyl ester in compound **11**, β-alanine ethyl ester in compound **17**, and aminoenanthic acid methyl ester in compound **19**. This differs them from GL conjugates that have residues in both the carbohydrate and triterpene regions (the compounds **2**–**8**) or dipeptides with aromatic amino acids (Tyr, Phe) (the compounds **21**–**23**). In compound **11,** L-glutamic acid dimethyl ester formed hydrogen bonds with His51, Asp75, and Gly153, while in compound **17**, β-alanine ethyl ester formed similar bonds with His51 and Asp75. Additionally, the C30-carboxy group in the triterpene part of both compounds **11** and **17** formed hydrogen bonds with Tyr150 in the same pattern. In compound **19**, the aminoethionic acid methyl ester formed a hydrogen bond with Gly153 (Figure 3B). The key residues identified as interacting with these three active compounds play a role in NS3pro substrate recognition [47]. The predicted interaction of NS3 protease with active compounds may be further validated through mutagenesis analysis in a future study. X-ray crystallographic studies revealed that the inhibitors bind to the hydrophobic catalytic pocket of dengue NS3 protease, stabilizing it in an open, inactive conformation [51]. Several small-molecule inhibitors targeting flaviviral NS2B-NS3 proteases have been discovered through virtual screening, with docking scores and hydrogen-bond interactions involving the catalytic triad (His51-Asp75-Ser135 in DENV-2) side chains [52,53].

Compounds **11**, **17**, and **19** were tested against DENV-2, showing concentration-dependent reductions in cytopathic effects, infectivity, and virus yields (Figure 5, Figure 6 and Figure 7). Compound 11 was the most potent, with EC50 values of 0.034 μM for infectivity, 0.042 μM for virus yield, and a selective index over 2000, aligning with its strong NS3 protease inhibition. Although compound 17 had better NS3 protease inhibition than compound 19, it showed weaker effects on infectivity and virus yield. At post-entry stage, all compounds strongly inhibited viral infectivity, but compound **19** also blocked viral entry (Figure 8). These findings emphasize the replication-stage activity of compounds **11** and **17**, with compound **19** acting on both entry and replication stages, explaining its higher efficacy in reducing infectivity and virus yield compared to compound 17. Notably, compound **19** shares structural similarity with the GL conjugated with valine methyl ester identified in our previous report, which inhibits DENV2 E-mediated attachment and is predicted to form hydrophobic interactions within a pocket at the interfaces of Domains I, II, and the stem region of the DENV2 envelope (E) protein [38]. Additionally, our previous studies found that modifying the GL molecule with amino acid residues enhanced its antiviral activity against DENV-2 [37,38]. The free C30-carboxy group in the triterpene part was essential for this effect. This study identified the active compounds as DENV-2 NS3 protease inhibitors: GL conjugates with L-glutamic acid dimethyl ester (compound **11**), β-alanine ethyl ester (compound **17**), and aminoenanthic acid methyl ester (compound **19**), all while keeping the triterpene part unchanged. These results align with our prior findings. Based on this study and our previous reports, compound **11** is the most potent GL derivative against DENV-2 and could be selected as a lead compound for further investigations of its antiviral activity in vitro and in vivo.

## 5. Conclusions

The study demonstrated that compound **11**, identified through computational screening as a DENV-2 NS3 protease inhibitor, exhibited the strongest inhibition of NS3 protease activity among all GL derivatives tested. It also showed significant antiviral activity against DENV-2, affecting in vitro infectivity, virus yield, and the post-entry stage. These findings underscore the effectiveness of computational methods in identifying potent DENV NS3 protease inhibitors, with compound **11** emerging as a lead candidate for the development of future antiviral agents against DENV infection.

## Figures and Tables

**Table 1:** Inhibition of NS2B-NS3 protease activity and DENV-2 infectivity by active compounds.

Compound Number	SMILES Code	NS2B-NS3 Protease ^a^ (IC_50_, μM)	Inhibition of DENV-2 Infectivity ^b^ (EC50, μM)	Cytotoxicity ^c^ (CC50, μM)	Selectivity Index (CC50/EC50)
**11**	C1C(C(C2[C@](C1)(C1[C@@](CC2)([C@]2(C(=CC1=O)C1[C@@](CC2)(CC[C@](C1)(C)C(=O)O)C)C)C)C)(C)C)O[C@@H]1OC([C@H]([C@@H](C1O[C@@H]1OC([C@H]([C@@H](C1O)O)O)C(=O)N[C@@H](C(=O)OC)CCC(=O)OC)O)O)C(=O)N[C@@H](C(=O)OC)CCC(=O)OC	0.0134 ± 0.002	0.034 ± 0.001	>100	>2941
**17**	C1C(C(C2[C@](C1)(C1[C@@](CC2)([C@]2(C(=CC1=O)C1[C@@](CC2)(CC[C@](C1)(C)C(=O)O)C)C)C)C)(C)C)O[C@@H]1OC([C@H]([C@@H](C1O[C@@H]1OC([C@H]([C@@H](C1O)O)O)C(=O)NCCC(=O)OCC)O)O)C(=O)NCCC(=O)OCC	0.34 ± 0.002	4.34 ± 0.008	>100	>23
**19**	C1C(C(C2[C@](C1)(C1[C@@](CC2)([C@]2(C(=CC1=O)C1[C@@](CC2)(CC[C@](C1)(C)C(=O)O)C)C)C)C)(C)C)O[C@@H]1OC([C@H]([C@@H](C1O[C@@H]1OC([C@H]([C@@H](C1O)O)O)C(=O)NCCCCCCC(=O)OC)O)O)C(=O)NCCCCCCC(=O)OC	0.52 ± 0.02	2.34 ± 0.08	>100	>42

^a^ A trans-cleavage FRET assay measured the derivatives’ inhibitory effects on DENV-2 NS2B/NS3 protease activity. IC50 is the concentration needed to inhibit 50% of enzymatic activity. ^b^ A 96 h DENV-2 infection in Vero E6 or A549 cells, assessed by IFA/DAPI assay, measured residual infectivity. EC50 is the concentration required to reduce infectivity by 50%. ^c^ CC50 is the concentration that reduces cell viability by 50%.

## Data Availability

No new data were created or analyzed in this study.

## Supplementary Materials

The following supporting information can be downloaded at: https://www.mdpi.com/article/10.3390/v16121926/s1, Figures S1–S25. HPLC for compounds **2**-**25**; Figures S26–S41. 1H NMR and 13C NMR for compounds **6**, **7**, **8**, **14**, **15**, **19**, **21**, **22**, and **23**. Figure S42. The cytotoxicity of active compounds was assessed on Vero E6 cells using the MTT assay.
