# Supplementary material for: Discovery of Potent Dengue Virus NS2B-NS3 Protease Inhibitors Among Glycyrrhizic Acid Conjugates with Amino Acids and Dipeptides Esters
Source: Viruses. 2024 Dec 17;16(12):1926. doi: 10.3390/v16121926 (PMC11680386; doi:10.3390/v16121926)
Supplement: Supplementary file 1 [file viruses-16-01926-s001.zip › viruses-3309458-supplementary.pdf]

SUPPLEMENTARY MATERIALS

Part I. Supplemental data of IR, ^{1}H NMR, and ^{13}C NMR for new compounds in the study

1. **IR spectra** (ν, cm^{-1}) **for the novel Glycyrrhizic acid derivatives**

Compound 6: 3600-3200 (OH, NH), 1738 (COOEt), 1663 (C=O), 1529 (CONH).
Compound 7: 3500-3200 (OH, NH), 1738 (COOMe), 1651 (C=O), 1535 (CONH).
Compound 8: 3500-3200 (OH, NH), 1739 (COOEt), 1661 (C=O), 1528 (CONH), 1498 (Ph).
Compound 14: 3600-3200 (OH, NH), 1738 (COOMe), 1658 (C=O), 1529 (CONH).
Compound 15: 3500-3200 (OH, NH), 1739 (COOMe), 1656 (C=O), 1533 (CONH).
Compound 19: 3500-3200 (OH, NH), 1734 (COOMe), 1656 (C=O), 1543 (CONH).
Compound 21: 3500-3200 (OH, NH), 1740 (COOMe), 1664 (C=O), 1661 (C=O), 1615 (Tyr), 1592 (CONH), 1518 (Tyr), 1500 (Tyr).
Compound 22: 3500-3200 (OH, NH), 1730 (COOMe), 1661 (C=O), 1652 (C=O), 1592 (CONH), 1560 (CONH), 1519, 1500 (Tyr).
Compound 23: 3600-3100 (OH, NH), 1740 (COOMe), 1668 (C=O), 1661 (C=O), 1591 (CONH), (CONH), 1500 (Ph).

2. *3-O-{2-O-[N-(β-D-glucopyranosyluronoyl)-L-leucine methyl ester]-N-(β-D-glucopyranosyluronoyl)-L-leucine methyl ester}-(3β, 20β)-11-oxo-30-(N-carbonyl-L-leucine methyl ester)-30-norolean-12-en**e 2* **[41].**
An amorphous solid, yield 80%. [α]$_D^{20}$ + 43° (c 0.1; EtOH). HPLC (97.5%, τ 2.68 min, Vidac) (Figure S1). Lit [41]: [α]$_D^{20}$ +40° (c 0.04; EtOH). IR (ν, cm-1): 3500-3200 (OH, NH), 1741 (COOMe), 1661 (C = O), 1531 (CONH). ^{1}H NMR (CD_3OD, δ, ppm): 8.10 (1H, br s, NH), 7.98 (2H, br s, 2NH), 5.61 (1H, s, H12), 4.67 (1H, d, *J* = 6.3 Hz, H1''), 4.55-4.54 (3H, m), 3.90-3.76 (3H, m), 3.72 (3H, s, OCH_3), 3.70 (2H, s), 3.68 (3H, s, OCH_3), 3.65 (3H, s, OCH_3), 3.64-3.58 (5H, m), 3.50-3.30 (6H, m), 3.15-3.13 (1H, m, H18), 2.74-2.38 (6H, m), 2.15-1.81 (8H, m), 1.70-1.52 (6H, m), 1.43 (3H, s, CH_3), 1.40-1.32 (2H, m), 1.27 (3H, s, CH_3), 1.24-1.20 (2H, m), 1.14 (3H, s, CH_3), 1.07 (2H, s), 1.05 (3H, s, CH_3), 0.96 (6H, s, 2CH_3), 0.95 (6H, s, 2CH_3), 0.94 (3H, s, CH_3), 0.92 (3H, s, CH_3), 0.90 (3H, s, CH_3), 0.87 (3H, s, CH_3), 0.82 (3H, s, CH_3), 0.70-0.67 (2H, m). ^{13}C NMR corresponded to published in [41]. Anal. calcd. for $C_{63}H_{101}N_3O_{19}$. C 62.82, H 8.37, N 3.49 %; found, C 62.70, H 8.27, N 3.42. M = 1204.45.

3. *3-O-{2-O-[N-(β-D-glucopyranosyluronoyl)-D-leucine methyl ester]-N-(β-D-glucopyranosyluronoyl)-D-leucine methyl ester}-(3β, 20β)-11-oxo-30-(N-carbonyl-D-leucine methyl ester)-30-norolean-12-ene **3*** **[42].**
An amorphous solid, yield 75%; [α]$_D^{20}$ +50° (c 0.04; EtOH). Lit [42]: [α]$_D^{20}$ +55° (c 0.04; EtOH). HPLC (96.2 %, τ 3.19 min, Kromasil) (Figure S2). IR (ν, cm-1): 3500-3200 (OH, NH), 1743 (COOMe), 1663 (C = O), 1530 (CONH). ^{1}H NMR (CD_3OD, δ, ppm): 7.95, 7.86 (3H, br s, 3NH), 5.60 (1H, s, H12), 4.72 (1H, d, *J* = 7.7 Hz, H1''), 4.56 (1H. d, J = 7.6 Hz, H1'), 4.53-4.49 (3H, m), 3.77-3.74 (3H, m), 3.72 (3H, s, OCH_3), 3.70 (3H, s, OCH_3), 3.68 (3H, s, OCH_3), 3.64-3.22 (10H, m), 3.20-3.19 (1H, m, H18), 2.99-2.53 (8H, m), 2.43 (1H, s, H9), 2.47-1.61 (6H, m), 1.54-1.45 (8H, m), 1.41 (3H, s, CH_3), 1.36 (2 H, s), 1.23 (3H, s, CH_3), 1.24-1.16 (2H, m), 1.13 (6H, s, 2CH_3), 1.06 (3H, s, CH_3), 1.06-0.98 (2H, m), 0.97 (3H, s, CH_3), 0.96 (3H, s, CH_3), 0.94 (3H, s, CH_3), 0.93 (3H, s, CH_3), 0.91 (3H, s, CH_3), 0.89 (3H, s, CH_3), 0.84 (3H, s, CH_3), 0.83 (3H, s, CH_3), 0.82-0.74 (2H, m).

^{13}C NMR spectra corresponded to published in [42]. Anal. calcd. for $C_{63}H_{101}N_3O_{19}$. C 62.82, H 8.37, N 3.49 %; found, C 62.70, H 8.25, N 3.35. M = 1204.45.

4. *3-O-{2-O-[N-(β-D-glucopyranosyluronoyl)-L-isoleucine methyl ester]-N-(β-D-glucopyranosyluronoyl)-L-isoleucine methyl ester}-(3β, 20β)-11-oxo-30-(N-carbonyl-L-isoleucine methyl ester)-30-norolean-12-ene* ***4*** **[41].**

   A white powder, yield 85%. $[\alpha]_D^{20}$ + 42° (c 0.04; EtOH). Lit [41]: $[\alpha]_D^{20}$ +40° (c 0.04; EtOH). HPLC (97.5%, τ 3.06 min, Vydac) (Figure S3); IR (ν, см$^{-1}$): 3500-3200 (OH, NH), 1742 (COOMe), 1661 (C=O), 1528 (CONH). ^1H NMR (500 MHz**,** CD_3OD, δ, ppm): 8.10 (1H, br s, N<u>H</u>), 7.44 (1H, d, 2N<u>H</u>), 5.71 (1H, s, H12), 4.60 (1H, d, *J* = 7.2 Hz, H1''), 4.50 (1H, d, *J* = 7.1 Hz, H1'), 4.46-4.39 (3H, m), 3.85-3.80 (3H, m), 3.73 (3H, s, OCH_3), 3.73 (3H, s, OCH_3), 3.72 (3H, s, OCH_3), 3.64-3.47 (8H, m), 3.34-3.23 (4H, m), 2.74-2.72 (1H, m, H18), 2.41 (1H, br s, H9), 2.24-2.15 (3H, m), 2.02-1.50 (14H, m), 1.47 (3H, s, CH_3), 1.27-1.23 (4H, m), 1.15 (3H, s, CH_3), 1.13 (6H, s, 2CH_3), 1.06 (3H, s, CH_3), 1.03-0.99 (2H, s), 0.96 (3H, s, CH_3), 0.95 (6H, s, 2CH_3)**,** 0.95 **(**3H, s, CH_3)**,** 0.93 (3H, s, CH_3), 0.91 (3H, s, CH_3), 0.89 (2H, s), 0.85 (3H, s, CH_3), 0.81 (3H, s, CH_3), 0.80-0.76 (2H, m). ^{13}C NMR spectra corresponded to published in [41]. Anal. calcd. for $C_{63}H_{101}N_3O_{19}$. C 62.82, H 8.37, N 3.49 %; found, C 62.72, H 8.25, N 3.40. M = 1204.45.

5. *3-O-{2-O-[N-(β-D-glucopyranosyluronoyl)-L-methionine methyl ester]-N-(β-D-glucopyranosyluronoyl)-L-methionine methyl ester}-(3β, 20β)-11-oxo-30-(N-carbonyl-L-methionine methyl ester)-30-norolean-12-ene* ***5*** **[41].**

   An amorphous solid, yield 78%. $[\alpha]_D^{20}$ +35° (c 0.04; EtOH). Lit [41]: $[\alpha]_D^{20}$ +30° (c 0.04; EtOH). HPLC (96.2%, τ 2.61 min, Vidac) (Figure S4). IR (ν, cm^{-1}): 3600-3200 (OH, NH), 1732 (COOMe), 1645 (C=O), 1521 (CONH). ^1H NMR (500 МГц, CD_3OD, δ, ppm): 8.10 (1H, s, N<u>H</u>), 7.88 (2H, s, 2N<u>H</u>), 5.62 (1H, s, H12), 4.64 (1H, d, *J* = 7.7 Hz, H1''), 4.51 (1H, d, *J* = 7.6 Hz, H1′), 3.84-3.78 (4H, m), 3.77 (3H, s, OCH_3), 3.74 (3H, s, OCH_3), 3.70 (3H, s, OCH_3), 3.60-3.46 (12H, m), 3.31-3.11 (5H, m), 2.85-2.53 (8H, m), 2.42 (1H, s, H9), 2.34-1.45 (18H, m), 1.41 (3H, s, CH_3), 1.37 (2H, s), 1.30-1.26 (3H, m), 1.24 (3H, s, CH_3), 1.13 (3H, s, CH_3), 1.12 (3H, s, CH_3), 1.06 (2H, s), 1.02 (3H, s, CH_3), 0.99-0.95 (2H, m), 0.83 (3H, s, CH_3), 0.78 (3H, s, CH_3), 0.76-0.74 (2H, m). ^{13}C NMR spectra corresponded to published in [41]. Anal. calcd. for $C_{60}H_{95}N_3O_{19}S_3$. C 57.25, H 7.61, N 3.34, S 7.64 %; found, C 57.12, H 7.52, N 3.22, S 7.50. M = 1258.56.

6. 3-O-{2-O-[N-(β-D-glucopyranosyluronoyl)-L-valine ethyl ester]-N-(β-D-glucopyranosyluronoyl)-L-valine ehyl ester}-(3β, 20β)-11-oxo-30-(N-carbonyl-L-valine ethyl ester)-30-norolean-12-ene **6**.

   An amorphous solid, yield 75%. $[\alpha]_D^{20}$ + 51° (*c* 0.04; EtOH). HPLC (95.8%, τ 2.79 min, Vidac) (Supplemental Figure S5). IR (ν, cm^{-1}): 3600-3200 (OH, NH), 1738 (COOEt), 1663 (C=O), 1529 (CONH). ^1H NMR (500 MHz, CD_3OD, *δ*, ppm.): 7.90 (1H, s, N<u>H</u>), 7.76 (1H, br s, N<u>H</u>), 7.70 (2H, br s, N<u>H</u>), 5.60 (1H, s, H12); 4.70 (1H, d, *J* = 7.7 Гц, H1''), 4.58 (1H, d, *J* = 7.7 Гц, H1′), 4.42-4.36 (3H, m), 4.22-4.18 (3H, m), 3.85-3.59 (6H, m), 3.55-3.43 (6H, m), 3.35-3.24 (4H, m), 2.86-2.54 (3H, m), 2.41 (1H, s, H9), 2.20-1.52 (18H, m), 1.44 (2H, s), 1.41 (3H, s, CH_3), 1.36-1.34 (2H, m), 1.30 (2H, s), 1.29 (3H, s, CH_3), 1.27 (3H, s, CH_3), 1.25-1.23 (2H, m), 1.18 (2H, s), 1.13 (3H, s, CH_3), 1.12 (3H, s, CH_3), 1.06 (3H. s, CH_3), 0.99 (3H, s, CH_3), 0.98 (6H, s, 2CH_3), 0.96 (3H, s, CH_3), 0.95 (3H, s, CH_3), 0.92 (2H, s), 0.84 (3H, s, CH_3), 0.83 (3H, s, CH_3), 0.78-0.75 (2H, m). ^{13}C NMR (125 MHz, CD_3OD, *δ*, м.д.): 200.90 (C11), 176.69 (C30), 170.12 (C13), 170.04 (C6''), 169.99 (C6'), 127.73 (C12), 103.67 (C1''), 103.60 (C1'), 89.02 (C3), 80.17 (C2'), 76.25 (C5''), 75.86 (C5'), 74.94 (C3''), 74.62 (C3'), 74.14 (C2''), 72.14 (C4'), 72.06

(C4''), 61.74 (C9), 55.06 (C5), 48.17 (C18), 45.36 (C8), 43.59 (C20), 43.18 (C14), 40.91 (C19), 39.33 (C4), 38.95 (C1), 37.46 (C22), 36.71 (C10), 32.42 (C7), 31.58 (C17), 30.94 (C21), 27.69 (C29), 27.60 (C28), 27.25 (C23), 27.15 (C2), 26.21 (C16), 26.02 (C15), 22.61 (C27), 18.19 (C26), 17.61 (C6), 17.10 (C25), 16.91 (C24); 3ValOEt: 173.00 (<u>C</u>OOEt), 171.15 (<u>C</u>OOEt), 170.93 (<u>C</u>OOEt), 61.11 (OCH_2), 61.01 (OCH_2), 60.90 (OCH_2), 57.60 (α-CH), 57.44 (α-CH), 57.08 (α-CH), 30.83 (CH), 30.74 (CH), 30.63 (CH), 18.19 (CH_3), 18.09 (CH_3), 18.00 (CH_3), 15.96 (CH_3), 15.80 (CH_3), 15.67 (CH_3), 13.33, 13.29 ($3CH_3$). Anal. calcd. for $C_{63}H_{101}N_3O_{19}$ C 62.28, H 8.45, N 3.48 %; found, C 62.13, H 8.25, N 3.28. M = 1204.45.

7. 3-O-{2-O-[N-(β-D-glucopyranosyluronoyl)-L-methionine ethyl ester]-N-(β-D-glucopyranosyluronoyl)-L-methionine ethyl ester}-(3β,20β)-11-oxo-30-(N-carbonyl-L-methionine ethyl ester)-30-norolean-12-ene **7**.

An amorphous solid, yield 76%; $[\alpha]_D^{20}$ +47°C (*c* 0.04, MeOH). HPLC (98.8±0.8%, τ 2.64 min, Vydac) (Supplemental Figure S6). IR (ν, $см^{-1}$): 3500-3200 (OH, NH), 1738 (COOMe), 1651 (C=O), 1535 (CONH). ^{1}H NMR (500 МГц, CD_3OD, δ, м.д.): 8.20, 7.85 (3H, s, 3N<u>H</u>), 5.60 (1H, s, H12), 4.62 (1H, d, *J* = 7.6 Hz, H1''), 4.49 (1H, d, J = 7.6 Hz), 3.83-3.77 (4H, m), 3.76, 3.74, 3.70 (6H, all s, $3OCH_2$), 3.60–3.46 (8H, m), 3.41-3.35 (3H, m), 3.31-3.26 (6H, m), 3.14-3.11 (2H, m), 2.85 (1H, s, H18), 2.68-2.65 (3H, m), 2.56-2.53 (2H, m), 2.42 (1H, s, H9), 2.36-2.01 (8H, m), 1.84-1.52 (14H, m), 1.41 (6H, br s, 2 CH_3), 1.37 (2H, br s), 1.32-1.28 (2H, m), 1.27 (3H, s, CH_3), 1.24-1.16 (2H, m), 1.13 (3H, s, CH_3), 1.12 (3H, s, CH_3), 1.06 (1H, br s), 1.02 (3H, s, CH_3), 0.97-0.89 (2H, m), 0.85 (3H, s, CH_3), 0.83-0.81 (2H, m), 0.79 (3H, s, CH_3), 0.76-0.74 (2H, m). ^{13}C NMR (CD_3OD, *δ*): 202.55 (C11), 175.79 (C30), 171.70 (C13), 171.16 (C6″), 170.70 (C6′), 129.11 (C12), 106.29 (C1″), 105.29 (C1′), 90.94 (C3), 84.00 (C2′), 77.35 (C5″), 77.10 (C5′), 76.34 (C3″), 76.17 (C3′), 73.20 (C2″), 72.97 (C4″), 72.83 (C4′), 63.13 (C9), 56.50 (C5), 49.51 (C18), 46.73 (C8), 44.90 (C20), 44.55 (C14), 42.43 (C19), 40.56 (C4), 40.22 (C1), 38.54 (C22), 38.03 (C10), 33.81 (C7), 32.97 (C17), 32.13 (C21), 29.69 (C29), 28.89 (C28), 27.59 (C23), 27.38 (C2), 27.04 (C16, C15), 23.80 (C27), 19.31 (C26), 18.42 (C6), 16.99 (C25), 16.64 (C24); 3MetOEt: 174.50 (<u>C</u>OOEt), 173.36 (<u>C</u>OOEt), 174.00 (<u>C</u>OOEt), 52.83 (α-CH), 52.69 (α-CH), 52.33 (α-CH), 28.40 (CH_2), 28.32 (CH_2), 28.17 ($2CH_2$), 16.99 (SCH_3), 16.64 ($2SCH_3$). Anal. calcd. for $C_{63}H_{101}N_3O_{19}S_3$ C 58.17, H 7.83, N 3.23, S 7.40%; found, C 57.82, H 7.75, N 3.10, S 7.28 %.

8. 3-O-{2-O-[N-(β-D-glucopyranosyluronoyl)-L-phenylalanine ethyl ester]-N-(β-D-glucopyranosyluronoyl)-L-phenylalanine ethyl ester}-(3β, 20β)-11-oxo-30-(N-carbonyl-L-phenylalanine ethyl ester)-30-norolean-12-ene **8**.

A white powder, yield 82%. $[\alpha]_D^{20}$ +49° (c 0.04; EtOH). HPLC (96.8%, τ 4.23 min, Pursuit) (Supplemental Figure S7). IR (ν, $см^{-1}$): 3500-3200 (OH, NH), 1739 (COOEt), 1661 (C=O), 1528 (CONH), 1498 (Ph). ^{1}H NMR (500 MHz, CD_3OD, ppm): 7.88 (1H, s, N<u>H</u>), 7.48 (2H, m, 2N<u>H</u>), 7.27-7.10 (15H, m, $3C_6H_5$), 5.64 (1H, s, H12), 4.80 (1H, d, *J* = 7.2 Hz, H1''), 4.75 (1H, d, *J* = 7.2 Hz, H1'), 4.13 (6H, br s), 3.72-3.30 (12H, m), 3.12-3.05 (6H, m), 2.82-2.32 (6H, m), 2.03-1.48 (18H, m), 1.35 (3H, br s, CH_3), 1.25 (2H, s), 1.18-1.16 (8H, m), 1.10 (3H, s, CH_3), 1.06 (3H, s, CH_3), 0.98 (3H, s, CH_3), 0.88-0.84 (2H, m), 0.82 (3H, s, CH_3), 0.76 (3H, s, CH_3), 0.78-0.75 (2H, m). C NMR (125 MHz, CD_3OD, δ, ppm): 201.01 (C11), 174.61 (C30), 170.39 (C13), 170.29 (C6″), 170.04 (C6′), 127.90 (C12), 103.46 (C1″), 102.94 (C1′), 89.48 (C3), 79.22 (C2′), 76.21 (C5″), 75.61 (C5′), 74.18 (C3″), 73.77 (C3′), 73.30 (C2″), 72.22 (C4″), 72.05 (C4′), 61.37 (C9), 55.09 (C5), 48.93 (C18), 45.46

(C8), 43.66 (C20), 43.30 (C14), 41.13 (C19), 39.49 (C4), 39.11 (C1), 37.62 (C22), 36.75 (C10), 32.60 (C7), 31.73 (C17), 31.06 (C21), 28.71 (C29), 28.33 (C28), 28.08 (C23), 27.55 (C2), 26.39 (C16), 26.22 (C15), 23.14 (C27), 18.50 (C26), 17.39 (C6), 16.34 (C25), 16.15 (C24); 3PheOEt: 171.93 (COOEt), 170.94 (COOEt), 170.81 (COOEt), 136.35 (C Ar), 135.89 (C Ar), 135.49 (C Ar), 129.25-128.35 (9C Ar), 128.08 (C Ar), 127.18 (C Ar), 127.05 (C Ar), 126.92 (C Ar), 126.80 (C Ar), 125.05 (C Ar), 61.78 (2OCH$_2$), 61.58 (OCH$_2$), 53.45 (α-CH), 53.37 (α-CH), 52.95 (α-CH), 37.48 (CH$_2$), 37.28 (CH$_2$), 37.09 (CH$_2$), 13.78 (CH$_3$), 13.74 (2CH$_3$). Anal. calcd. for $C_{75}H_{101}N_3O_{19}$ C 66.79, H 7.55, N 3.11%, found, C 66.65, H 7.38, N 3.00%. M = 1348.57.

9. 3-O-{2-O-[N-(β-D-glucopyranosyluronoyl)-L-tryptophan methyl ester]-N-(β-D-glucopyranosyluronoyl)-L-tryptophan methyl ester}- (3β, 20β)-11-oxo-30-noroleane-12-ene-30-oic acid **14.**

An amorphous solid, yield 55%; $[\alpha]_D^{20}$ +65° (c 0.08, EtOH). HPLC (95.9%, τ 3.32 min, Hypersil) (Supplemental Figure S13). IR (ν, cm^{-1}): 3600-3200 (OH, NH), 1738 (COOMe), 1658 (C=O), 1529 (CONH). ^{1}H NMR (500 МГц, CD$_3$OD, *δ*, м.д.): 7.95 (1H, br s, NH), 7.65 (1H, br s, NH), 7.53-7.50 (2H, m, 2Ar-H), 7.44-7.40 (4H, m, 4Ar-H), 7.31 (2H, d, J = 8 Hz, 2Ar-H), 7.08 (6H, m, 6Ar-H), 7.00-6.98 (2H, m, Ar-H), 5.56 (1H, s, H12), 4.76 (1H, d, J = 7.2, H1″), 4.69 (2H, m), 4.54 (1H, d, J = 7.2, H1′), 3.86-3.28 (6H, m), 3.66 (3H, s, OCH$_3$), 3.61 (3H, s, OCH$_3$), 3.63 (1H, s), 3.59-3.43 (9H, m), 3.33-3.30 (6H, m), 3.20-3.12 (3H, m), 2.62-2.52 (3H, m), 2.3 (1H, s, H9), 2.17-1.47 (15H, m), 1.35 (4H, br s, 2CH$_2$), 1.31 (3H, s, CH$_3$), 1.22-1.17 (2H, m), 1.14 (3H, s, CH$_3$), 1.12-1.08 (2H, m), 1.04 (3H, s, CH$_3$), 1.01 (6H, s, 2CH$_3$), 0.98-0.84 (4H, m), 0.79 (3H, s, CH$_3$), 0.77 (3H, s, CH$_3$), 0.67-0.64 (2H, m). ^{13}C NMR (125 MHz, CD$_3$OD, *δ*, ppm): 202.54 (C11), 180.54 (C30), 172.85 (C13), 171.58 (C6"), 171.46 (C6'), 128.61 (C12), 104.85 (C1"), 103.96 (C1'), 91.33 (C3), 78.98 (C2'), 77.82(C5"), 77.25 (C5'), 76.06 (C3"), 75.56 (C3'), 74.85 (C2″), 73.36 (C4"), 73.16 (C4'), 62.99 (C9), 56.37 (C5), 49.80 (C18), 46.64 (C8), 44.88 (C20), 44.48 (C14), 42.36 (C19), 40.56 (C4), 40.15 (C1), 38.96 (C22), 37.91 (C10), 33.67 (C7), 32.88 (C17), 31.99 (C21), 29.20 (C29), 28.83 (C28), 28.19 (C23), 27.48 (C2), 27.29 (C16), 27.06 (C15), 23.86 (C27), 19.28 (C26), 18.35 (C6), 17.34 (C25), 17.03 (C24); 2TrpOMe: 173.54 (COOMe), 173.21 (COOMe), 138.20 (C Ar), 137.89 (C Ar), 128.84 (C Ar), 128.78 (C Ar), 126.27 (C Ar), 124.42 (C=), 122.53 (C=), 122.42 (C=), 119.96 (C Ar), 119.80 (C Ar), 119.22 (C Ar), 112.83 (C Ar), 112.53 (C Ar), 110.13 (C Ar), 108.82 (C Ar), 54.66 (α-CH), 53.45 (α-CH), 52.97 (2OCH$_3$), 28.31 (2CH$_2$). Anal. calcd. for $C_{66}H_{86}N_4O_{20}$ C 63.14; H 6.90; N 4.46%; found, C 62.95, H 6.88, N 4.32%. M = 1255.37.

10. 3-O-{2-O-[N-(β-D-glucopyranosyluronoyl)-L-methionine ethyl ester]-N-(β-D-glucopyranosyluronoyl)-L-methionine ethyl ester}-(3β,20β)-11-oxo-30--norolean-12-ene30-oic acid **15**.

An amorphous solid, yield 54% HPLC (98.8%, τ 2.74 min, Vidac) (Supplemental Figure S14). $[\alpha]_D^{20}$ +58°C (*c* 0.04, EtOH). IR (ν, см$^{-1}$): 3500-3200 (OH, NH), 1739 (COOMe), 1656 (C=O), 1533 (CONH). ^{1}H NMR (500 MHz, CD$_3$OD, δ, ppm): 8.10, 7.88 (2H, br s, 2NH), 5.62 (1H, s, H12), 4.64 (1H, d, *J* = 7.5 Hz, H1’’), 4.57-4.52 (2H, m), 4.51 (1H, d, *J* = 7.5 Hz), 4.23-4.13 (3H, m), 3.84-3.80 (4H, m), 3.76 (2H, s, OCH$_2$), 3.74 (2H, br s, OCH$_2$), 3.68–3.38 (6H, m), 3.31 (3H, br s), 3.28-3.10 (4H, m), 2.85 (1H, s), 2.68-2.52 (2H, m), 2.42 (1H, s, H9), 2.18-1.45 (14H, m), 1.42 (6H, br s, 2 CH$_3$), 1.36 (2H, s), 1.30-1.29 (2H, m), 1.28 (3H, s, CH$_3$), 1.27 (3H, s, CH$_3$), 1.25-1.16 (3H, m), 1.13 (6H, br s, 2CH$_3$), 1.02 (2H, s, CH$_2$), 0.98-0.88 (2H, m), 0.86 (3H, s, CH$_3$), 0.85-0.83 (2H, m), 0.79 (3H, s, CH$_3$), 0.75-0.73

(2H, m). ^{13}C NMR (125 MHz, CD_3OD, δ, ppm): 8.12, 7.88 (2H, s, 2N<u>H</u>), 5.60 (1H, s, H12), 4.64 (1H, d, *J* = 7.6 Hz, H1''), 4.54 (1H, d, J = 7.6 Hz), 4.51-4.50 (1H, m), 3.86-3.78 (3H, m), 3.77 (2H, br s, OCH_2), 3.74 (2H, br s, OCH_2), 3.69–3.65 (2H, m), 3.60-3.48 (3H, m), 3.44-3.34 (6H, m), 3.31 (4H, br s), 3.29-3.13 (3H, m), 2.68-2.50 (4H, m), 2.44 (1H, s, H9), 2.22-1.46 (10H, m), 1.42 (6H, br s, 2 CH_3), 1.37 (2H, br s), 1.33-1.28 (4H, m), 1.27 (3H, s, CH_3), 1,24-1.20 (2H, m), 1.16 (2H, s), 1.13 (6H, br s, 2CH_3), 1.09 (2H, s), 1.02 (3H, s, CH_3), 0.98-0.89 (2H, m), 0.86 (3H, s, CH_3), 0.85-0.81 (2H, m), 0.77 (3H, s, CH_3), 0.72-0.70 (2H, m). ^{13}C NMR (CD_3OD, *δ*): 202.55 (C11), 182.00 (C30), 171.78 (C13), 170.71 (C6″), 170.40 (C6′), 129.17 (C12), 106.27 (C1″), 105.29 (C1′), 90.94 (C3), 83.97 (C2′), 77.34 (C5″), 77.07 (C5′), 76.34 (C3″), 76.17 (C3′), 73.10 (C2″), 72.96 (C4″), 72.82 (C4′), 63.12 (C9), 56.49 (C5), 49.91 (C18), 46.72 (C8), 45.07 (C20), 44.51 (C14), 42.35 (C19), 40.56 (C4), 40.21 (C1), 38.32 (C22), 38.02 (C10), 32.98 (C7), 32.13 (C17), 30.72 (C21), 28.40 (C29), 28.16 (C28), 27.58 (C23), 27.38 (C2), 26.55 (C16), 26.09 (C15), 23.74 (C27), 19.31 (C26), 18.42 (C6), 16.98 (C25), 16.64 (C24); 2MetOEt: 173.26 (<u>C</u>OOEt), 172.20 (<u>C</u>OOEt), 52.70 (α-CH), 52.53 (α-CH), 33.87 (CH_2), 33.80 (CH_2), 28.88 (CH_2), 28.81 (CH_2), 14.49 (SCH_3), 14.41 (SCH_3). Anal. calcd. for $C_{56}H_{88}N_2O_{18}S_2$ C 58.92, H 7.77, N 2.45, S 5.62 %; found, C 58.80, H 7.65, N 2.40, S 5.45%. M = 1141.43.Anal. calcd. for $C_{54}H_{88}O_{18}N_2S_2$ C 58.92, H 7.77, N 2.45; S 5.62; found, C 58.84, H 7.58, N 2.46, S 5.64%. M = 1141.43.

11. 3-O-{2-O-[N-(β-D-glucopyranosyluronoyl)-ω-aminoenanthic acid methyl ester]-N-(β-D-glucopyranosyluronoyl)-ω-aminoethantic acid methyl ester}- (3β, 20β)-11-oxo-30-noroleane-12-ene-30-acid **19**.

An amorphous solid, yield 52%; $[\alpha]_D^{20}$ +55° (c 0.06, EtOH). HPLC (97.8%, τ 2.62 min, Pursuit) (Supplemental Figure S18). IR (ν, cm^{-1}): 3500-3200 (OH, NH), 1734 (COOMe), 1656 (C=O), 1543 (CONH). ^{1}H NMR (500 CD_3OD, δ, ppm): 7.90 (2H, s, 2N<u>H</u>), 5.58 (1H, s, H12), 4.72 (1H, d, *J* = 7.2 Hz, H1''), 4.64 (1H, d, *J* = 7.2 Hz, H1'), 4.56-4.52 (2H, m), 4.42-4.08 (4H, m), 3.76-3.65 (4H, m), 3.75 (3H, s, OCH_3), 3.70-3.64 (2H, m), 3.64 (3H, s, OCH_3), 3.59-3.21 (12H, m), 2.88-2.68 (4H, m), 2.58-2.56 (4H, m), 2.42-1.53 (18H, m), 1.45 (3H, s, CH_3), 1.36 (3H, s, CH_3), 1.35-1.22 (11H, m), 1.16 (3H, s, CH_3), 1.12 (6H, s, 2CH_3), 1.06-0.86 (8H, m), 0.83 (6H, s, 2CH_3), 0.79-0.75 (2H, m). ^{13}C NMR (125 MHz, CD_3OD, δ, ppm): 201.18 (C11), 179.04 (C30), 170.48 (C13), 169.00 (C6'', C6'), 127.57 (C12), 104.80 (C1''), 103.90 (C1'), 89.42 (C3), 82.50 (C2'), 75.96 (C5''), 75.70 (C5'), 74.89 (C3''), 74.65 (C3'), 74.54 (C2''), 72.16 (C4''), 71.67 (C4'), 61.77 (C9), 55.17 (C5), 48.54 (C18), 45.39 (C8), 43.54 (C20), 43.25 (C14), 41.04 (C19), 38.88 (C4), 38.60 (C1), 37.66 (C22), 36.67 (C10), 32.46 (C7), 31.62 (C17), 30.66 (C21), 27.89 (C29), 27.53 (C28), 27.05 (C23), 27.05 (C2), 26.24 (C16), 26.03 (C15), 22.26 (C27), 18.00 (C26), 17.08 (C6), 15.71 (C25), 15.54 (C24); $2NH(CH_2)_6COOCH_3$: 171.70 (COOMe), 171.44 (COOMe), 51.43 (OCH_3), 51.00 (OCH_3), 39.32 (CH_2), 39.22 (CH_2), 33.58 (CH_2), 33.45 (CH_2), 29.07 (CH_2), 28.77 (CH_2), 28.65 (CH_2), 28.52 (CH_2), 26.95 (CH_2), 26.86 (CH_2), 24.74 (CH_2), 24.63 (CH_2). Anal. calcd. for $C_{58}H_{92}N_2O_{18}$. C 63.02, H 8.39, N 2.53 %; found, C 62.88, H 8.25, N 2.46. M = 1105.33.

12. 3-O-{2-O-[N-(β-D-glucopyranosyluronoyl)-glycyl-L-tyrosine methyl ester]-N-(β-D-glucopyranosyluronoyl)-glycyl-L-tyrosine methyl ester}-(3β,20β)-11-oxo-30-noroleane-12-ene-30-acid **21**

To a solution of GL (0.82 g, 1 mmol) in DMF (20 ml) N-ethylmorpholine (0.7 ml, 6 mmol) and a Woodward's reagent K (0.63 g, 2.5 mmol) were added at 0-5°C. A mixture was stirred at this temperature for 1.5 h and at 20-22 °C for 1.5 h. Then N-ethylmorpholine (0.5 ml) and CF_3COOH • Gly-TyrOMe (0.92 g, 2.5 mmol) were added and a reaction mixture was kept

at 20-22 °C for 48 h with a periodic stirring. Then it was evaporated to dryness and a residue was purified on a silica gel column using a gradient mixture $CHCl_3$-MeOH-H_2O (400:10:1→50:10:1, v%) with TLC control. Compound **21** was isolated as an amorphous solid, yield 55%. HPLC (98.5%, τ 2.42 min, Zorbax) (Supplemental Figure S21). $[\alpha]_D^{20}$ +56° (*c* 0.1, MeOH). IR (*ν*, cm^{-1}): 3500-3200 (OH, NH), 1740 (COOMe), 1664 (C=O), 1661 (C=O), 1615 (Tyr), 1592 (CONH), 1518 (Tyr), 1500 (Tyr). ^{1}H NMR (500 МГц, CD_3OD, δ): 8.42 (2H, s, 2H), 8.08 (4H, d, *J* = 7.6 Hz, 4H Ar), 7.90-7.85 (2H, m, 2NH), 7.61-7.58 (2H, m, 2H Ar), 7.05-7.00 (2H, m, 2NH), 6.72 (2H, s, 2H Ar), 5.57 (1H, s, H12), 4.68 (1H, d, *J* = 7.6 Hz, H1″), 4.56 (1H, d, J = 7.6 Hz, H1'), 4.00-3.92 (3H, m), 3.81 (6H, br s, 2 OCH_3), 3.76-3.45 (6H, m), 3.34 (2H, s), 3.30-3.18 (6H, m), 3.12-2.66 (3H, m), 2.62 (1H, s, H9), 2.41-1.55 (12H, m), 1.38 (6H, s, 2CH_3), 1.33-1.30 (5H, m), 1.28-1.23 (2H, m), 1.16 (6H, br s, 2CH_3), 1.13 (6H, s, 2CH_3), 1.05 (2H, m), 1.00-0.98 (2H, m), 0.81 (3H, s, CH_3), 0.78-0.75 (2H, m). ^{13}C NMR (MHz, CD_3OD, *δ*): 201.21 (C11), 179.06 (C30), 169.20 (C13), 167.78 (C6″), 167.20 (C6′), 127.51 (C12), 103.75 (C1″), 103.65 (C1′), 89.29 (C3), 81.60 (C2′), 76.20 (C5″), 75.80 (C5′), 74.69 (C3″), 74.51 (C3′), 72.17 (C2″), 71.97 (C4″), 71.81 (C4′), 61.73 (C9), 56.61 (C5), 48.86 (C18), 45.37 (C8), 43.54 (C20), 43.35 (C14), 41.03 (C19), 40.16 (C4), 39.30 (C1), 37.66 (C22), 36.64 (C10), 32.39 (C7), 31.61 (C17), 30.65 (C21), 27.91 (C29), 27.49 (C28), 27.02 (C23), 26.20 (C16), 26.00 (C15), 25.60 (C2), 22.59 (C27), 18.00 (C26), 17.10 (C6), 15.78 (C25), 15.65 (C24); 2Gly-TyrOMe: 171.84 ($COOCH_3$), 171.59 ($COOCH_3$), 166.48 (CONH), 165.93 (CONH), 156.12 (C Ar), 155.98 (C Ar), 130.56 (C Ar), 130.16 (C Ar), 130.06 (C Ar), 129.99 (C Ar), 129.93 (C Ar), 128.89 (C Ar), 128.77 (C Ar), 125.43 (C Ar), 125.32 (C Ar), 115.04 (C Ar), 115.00 (C Ar), 66.40 (CH_2), 66.11 (CH_2), 54.88 (α-CH), 54.38 (α-CH), 51.53 (OCH_3), 51.26 (OCH_3), 38.21 (CH_2), 38.05 (CH_2). Anal. calcd. for $C_{66}H_{90}N_4O_{22}$, C 61.38; H 7.02; N 4.34%; found, C 61.22; H 6.90; N 4.24. M = 1291.40.

13. 3-O-{2-O-[N-(β-D-glucopyranosyluronoyl)-L-isoleucine-tyrosine methyl ester)]-N-(β-D-glucopyranosyluronoyl)-L-isoleucine-tyrosine methyl ester)}-(3β,20β)-11-oxo-30-noroleane-12-ene-30-oic acid **22.**.

To a solution of GL (0.82 g, 1 mmol) in DMF (20 ml) N-ethylmorpholine (0.7 ml, 6 mmol) was and Woodward's reagent K (0.6 г, 2.5 ммоль) were added at 0-5 ° C and stirred at 0-5°C for 1.5 h and at 20-22 °C for 1.5 h. Then N-ethylmorpholone (0.7 ml) и CF_3COOH•Ile-TyrOMe (1.12 g, 3 mmol) were added and a reaction was kept at 20-22 °C for 48 h with a periodic stirring. Then it was evaporated and the residue was subjected to column cromatography as described for **21.** The compound **22** was isolated as an amorpous solid, yield 54%; $[\alpha]_D^{20}$ +49° (*c* 0.04, EtOH). IR (ν, sm^{-1}): 3500-3200 (OH, NH), 1730 (COOMe), 1661 (C=O), 1652 (C=O), 1592 (CONH), 1560 (CONH), 1519, 1500 (Tyr). HPLC (96.0%, τ 2.56 min, the Vydac) (Supplemental Figure S23). ^{13}C NMR (125 МГц, DMSO-d_6, *δ*, м.д.) : 199.64 (C11), 178.48 (C30), 170.43 (C13), 169.00 (C6"), 168.70 (C6'), 128.33 (C12), 104.40 (C1"), 103.92 (C1'), 89.70 (C3), 82.70 (C2′), 76.34 (C5″), 76.13 (C5'), 75.90 (C3'), 75.31.C3"), 72.60 (C2"), 72.13 (C4"), 71.80 (C4'), 61.51 (C9), 54.69 (C5), 48.47 (C18), 45.31 (C8), 43.54 (C20), 43.30 (C14), 41.15 (C19), 40.16 (C4), 39.17 (C1), 37.93 (C22), 36.73 (C10), 32.54 (C7), 31.93 (C17), 30.89 (C21), 28.80 (C29), 28.34 (C28), 27.62 (C23), 27.45 (C2), 26.49 (C16), 26.18 (C15), 23.37 (C27), 18.76 (C26), 17.34 (C25), 16.40 (C24); 2Ile-TyrOMe: 171.80 ($COOCH_3$), 171.60 ($COOCH_3$), 166.80 (CONH), 166.70 (CONH), 156.10 (C Ar), 156.00 (C Ar), 130.79 (C Ar), 130.57 (C Ar), 130.49 (C Ar), 130.12 (C Ar), 129.28 (C Ar), 128.94 (C Ar), 128.33 (C Ar), 127.67 (C Ar), 127.00 (C Ar), 115.46 (C Ar), 115.34 (C Ar), 57.10 (α-CH), 56.80 (α-CH), 55.30 (α-CH), 55.00 (α-CH), 51.91 (OCH_3),

51.82 (OCH_3), 37.50 (CH), 37.60 (CH), 34.10 (CH_2), 33.78 (CH_2), 26.18 (CH), 26.05 (CH), 14.99 ($2CH_3$), 11.80 (CH_3), 11.59 (CH_3). Anal. calc. for $C_{74}H_{106}N_4O_{22}$ C 63.32, H 7.61, N 3.99%; found, C 63.15, H 7.53, N 3.90%. M = 1403.61.

14. 3-O-{2-O-[N-(β-D-glucopyranosyluronoyl)-L-isoleucine-phenylanine methyl ester)]-N-(β-D-glucopyranosyluronoyl)-L-isoleucine-phenylalanine methyl ester)}-(3β,20β)-11-oxo-30-noroleane-12-ene-30-oic acid **23**.
It was isolated as an amorphous solid, yield 52%. HPLC (96.0%, τ 3.24 min, Discovery) (Supplementary Figure S25). $[\alpha]_D^{20}$ +52° (c 0.1, MeOH). IR, ν, cm^{-1}: 3500-3100 (OH, NH), 1740 (COOMe), 1668, 1661, 1591, 1570, 1500 (Ph). ^{13}C NMR: (125 МГц, DMSO-d_6, *δ*, м.д.):199.65 (C11), 178.30 (C30), 170.40 (C13), 169.79 (C6″), 169.20 (C6′), 128.18 (C12), 104.12 (C1″), 103.77 (C1′), 88.89 (C3), 83.35 (C2′), 76.26 (C5″), 75.78 (C5′), 75.21 (C3″), 75.10 (C3′), 72.77 (C2′), 71.74 (C4″), 71.62 (C4′), 61.63 (C9), 54.99 (C5), 48.23 (C18), 45.30 (C8), 43.46 (C20), 43.14 (C14), 41.15 (C19), 39.33 (C4), 39.05 (C1), 37.82 (C22), 36.66 (C10), 32.59 (C7), 31.82 (C17), 30.99 (C21), 28.68 (C29), 28.42 (C28), 27.58 (C23), 27.38 (C2), 26.39 (C16), 26.29 (C15), 23.88 (C27), 18.66 (C26), 17.23 (C25), 16.44 (C24); 2Ile-PheOMe: 172.11 (COOMe), 171.83 (COOMe), 168.85 (CONH), 168.76 (CONH), 137.04 (C Ar), 136.70 (C Ar), 129.54 (C Ar), 129.28 (2C Ar), 128.44 (2C Ar), 128.18 (C Ar), 127.97 (C Ar), 127.60 (C Ar), 126.85 (C Ar), 126.76 (C Ar), 56.70 (α-CH), 56.50 (α-CH), 53.80 (α-CH), 53.72 (α-CH), 52.12 (OCH_3), 51.99 (OCH_3), 37.40 (CH), 37.30 (CH), 37.18 (CH_2), 37.00 (CH_2), 24.20 (CH), 23.88 (CH), 15.45 (CH_3), 15.33 (CH_3), 13.90 (CH_3), 13.82 (CH_3). Anal. calc. for $C_{74}H_{106}N_4O_{20}$, C 64.79, H 7.79, N 4.08%; found, C 64.66, H 7.70, N 3.95%. M = 1371.61.

Part II. Supplemental figures for HPLC analysis data of GL derivatives

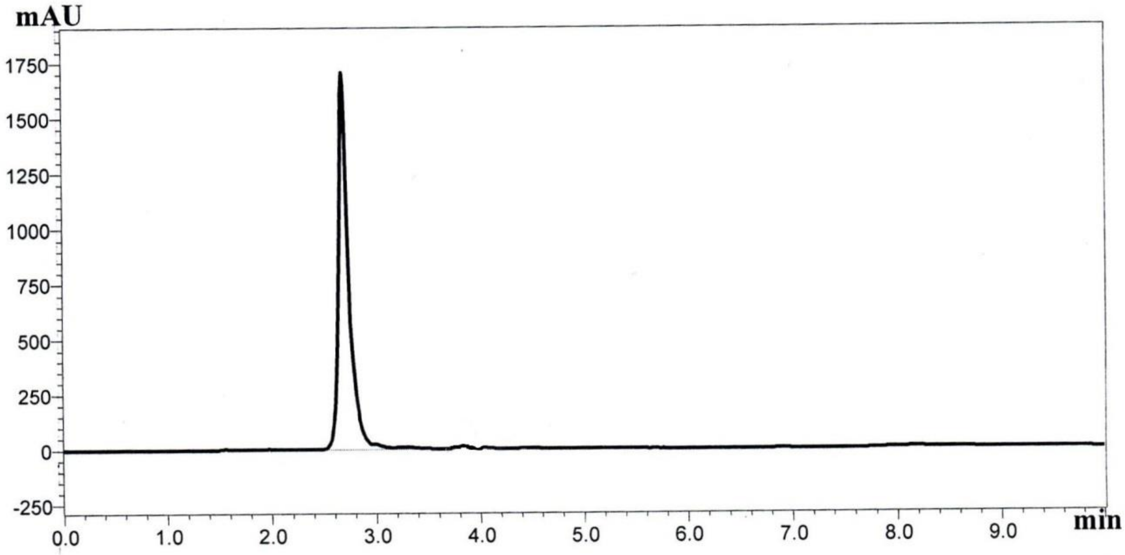

Figure S1. HPLC for compound **2**. The Vydac TPC18 column, mobile phase CH_3OH; the flow rate 1.0 ml/min; the retention time was 2.68 min; UV detection was carried out at λ 254 nm; the purity was 97.5±0.8%.

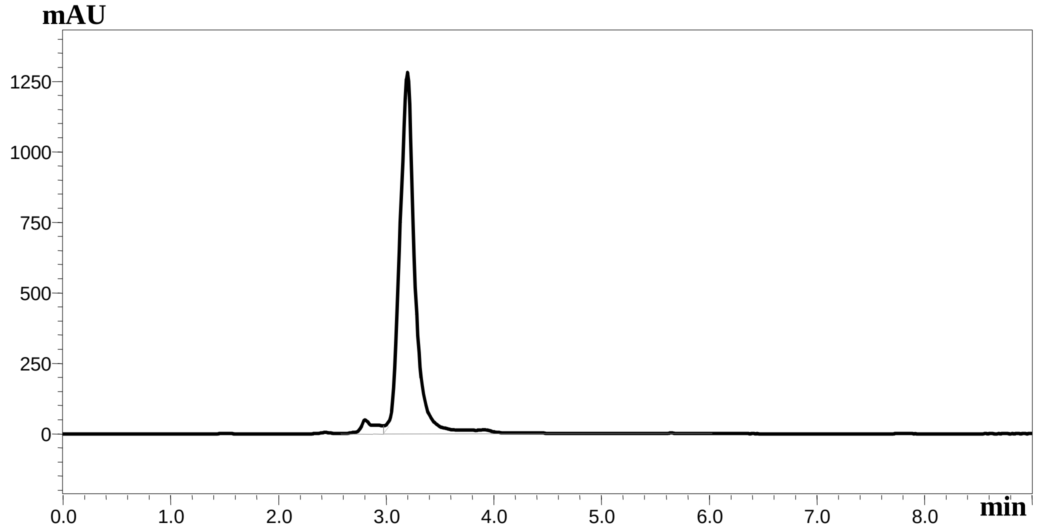

Figure S2. HPLC for compound **3.** The Kromasil 218 C18 column, mobile phase CH_3OH; the flow rate 1.0 ml/min; the retention time was 3.19 min; UV detection was carried out at λ 254 nm; the purity was 96.2±0.8%.

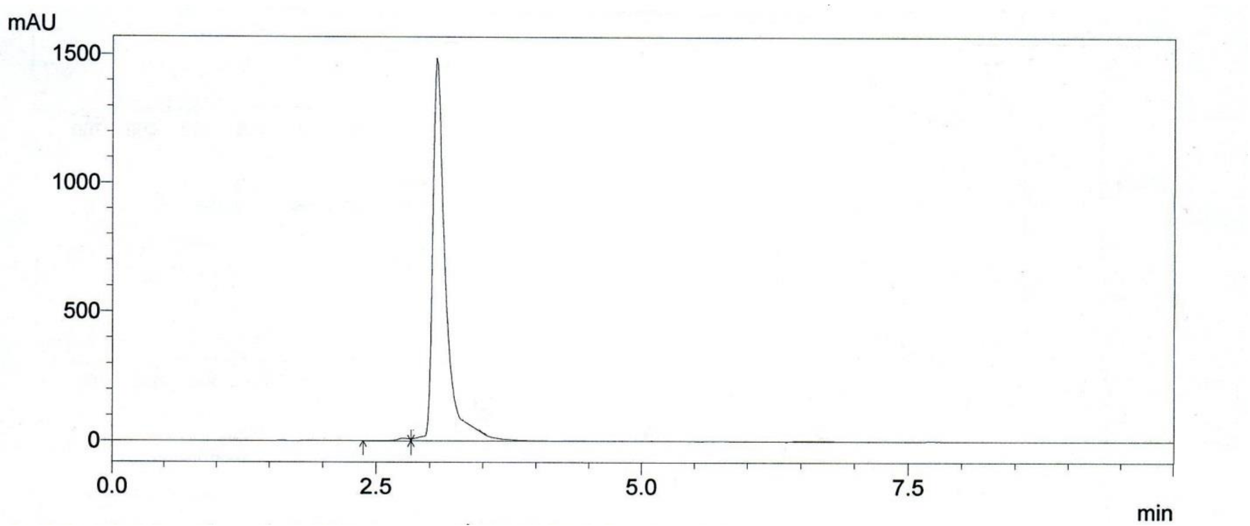

Figure S3. HPLC for compound **4.** The Vydac TPC18(1-3) column, mobile phase CH_3OH; the flow rate 1.0 ml/min; the retention time was 3.06 min; UV detection was carried out at λ 254 nm; the purity was 97.5±0.8%.

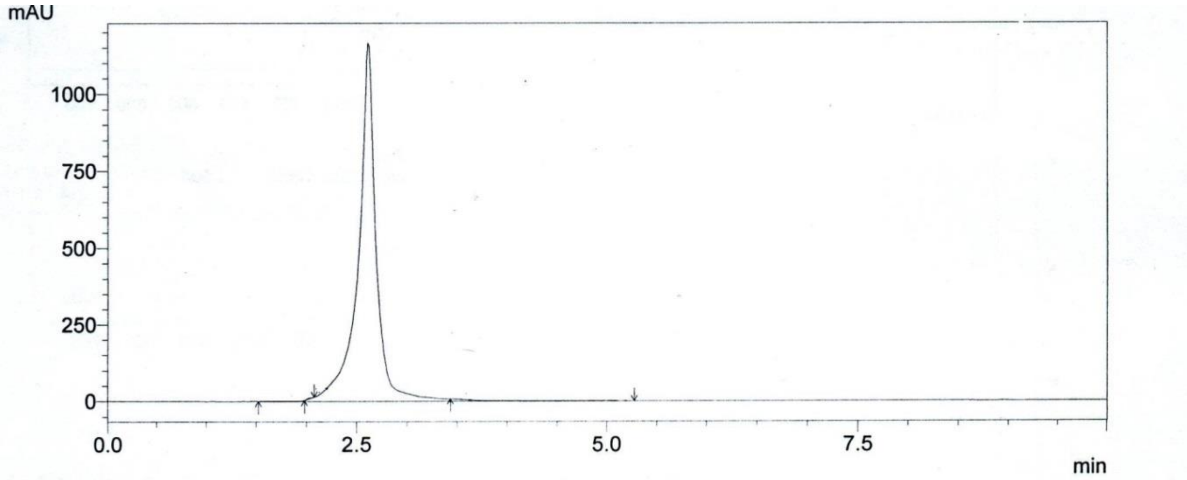

Figure S4. HPLC for compound **5.** The Vydac TPC18(1-3) column, mobile phase CH_3OH; the flow rate 1.0 ml/min; the retention time was 2.61 min; UV detection was carried out at λ 254 nm; the purity was 96.5±0.8%.

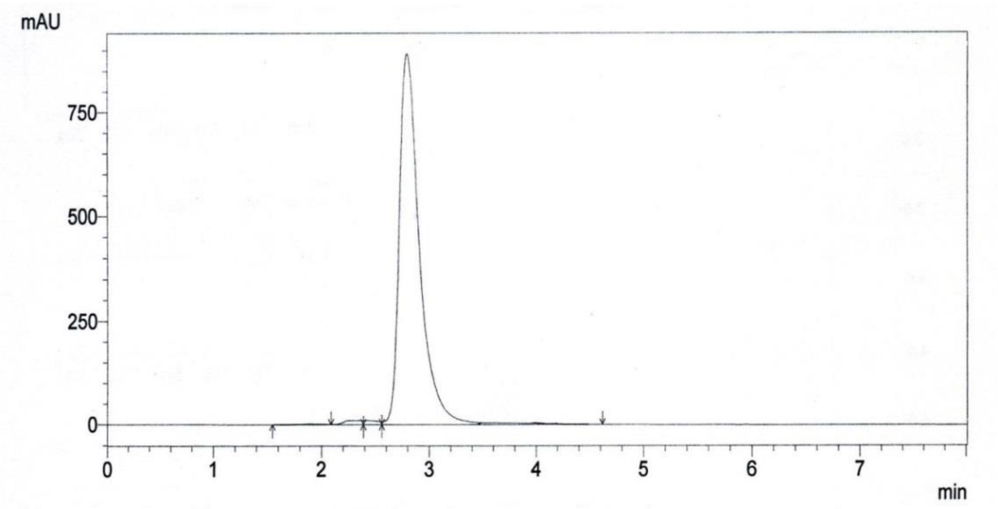

Figure S5. HPLC for compound **6.** Vidac TPC18 column, mobile phase CH_3OH; the flow rate 1.0 ml/min; the retention time was 2.79 min; UV detection was carried out at λ 254 nm; the purity was 95.8±0.8%.

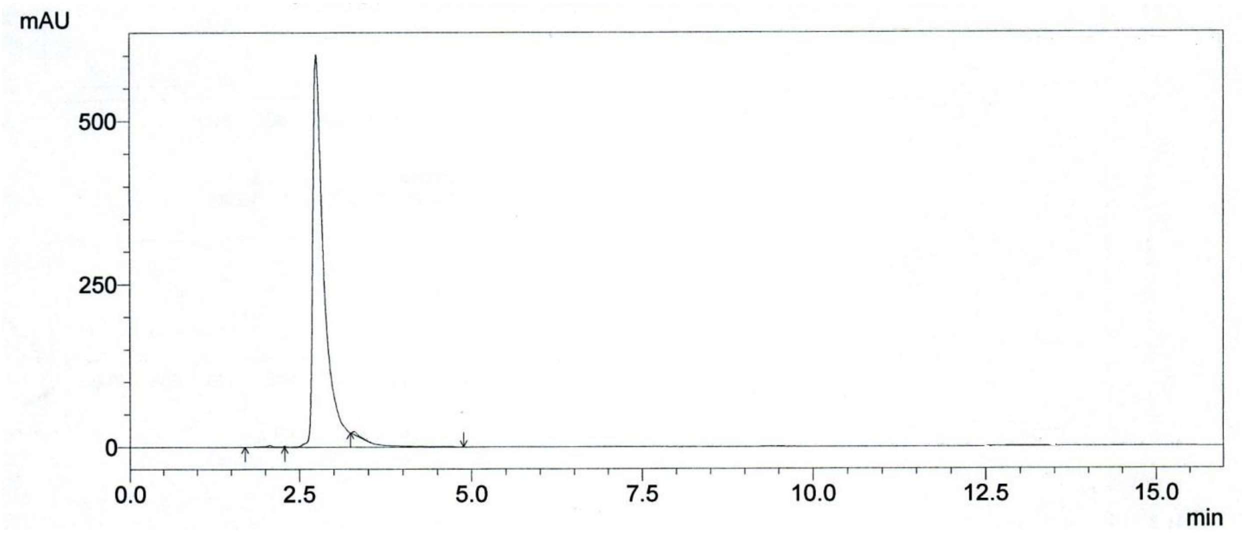

Figure S6. HPLC for compound **7**. The Vidac TPC18 column, mobile phase CH_3OH; the flow rate 1.0 ml/min; the retention time was 2.64 min; UV detection was carried out at λ 254 nm; the purity was 98.8±0.8%.

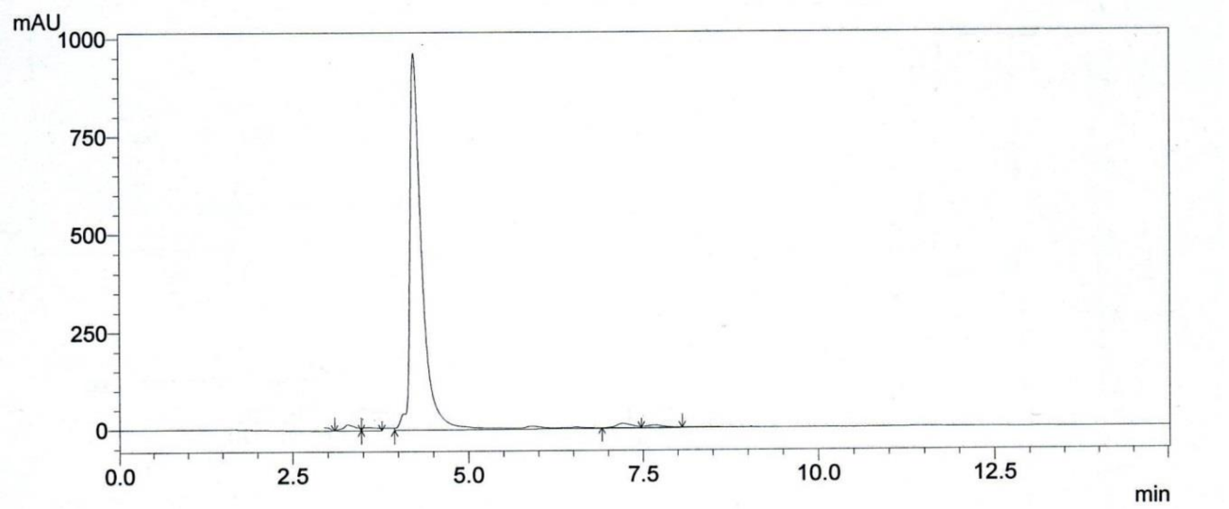

Figure S7. HPLC for compound **8.** Pursuit C18 column, mobile phasr CH_3OH; the flow rate 1.0 ml/min; the retention time was 4.23 min; UV detection was carried out at λ 254 nm; the purity was 96.8±0.8%.

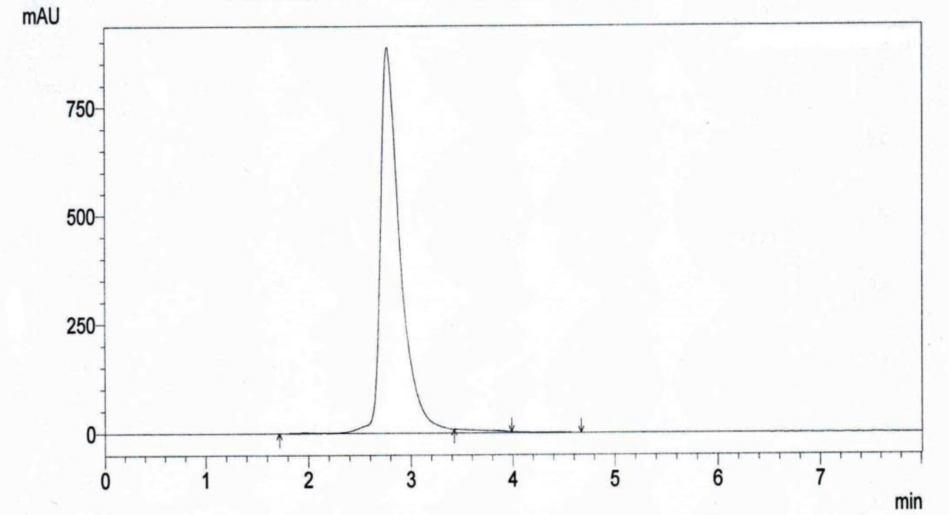

Figure S8. HPLC for compound **9**. The Vydac TP C18(1-3); mobile phase CH_3OH; the flow rate 1.0 ml/min; the retention time was 2.78 min; UV detection was carried out at λ 254 nm; the purity was 97.5±0.8%.

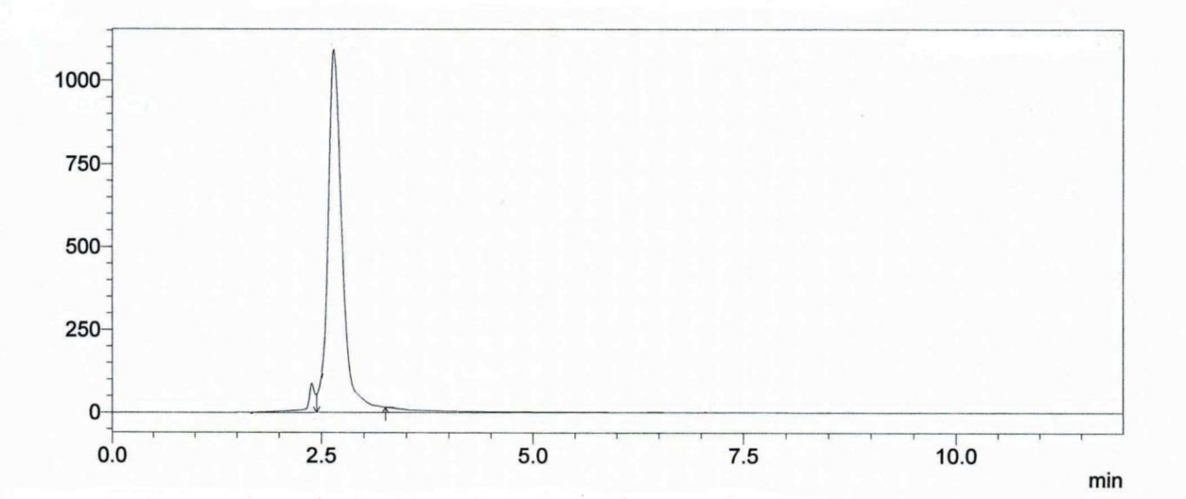

Figure S9. HPLC for compound **10.** A Vydac TP C18 column; a mobile phase CH_3OH; a flow rate 1.0 ml/min; a retention time was 2.65 min; UV detection was carried out at λ 254 nm; the purity was 96.5±0.8%.

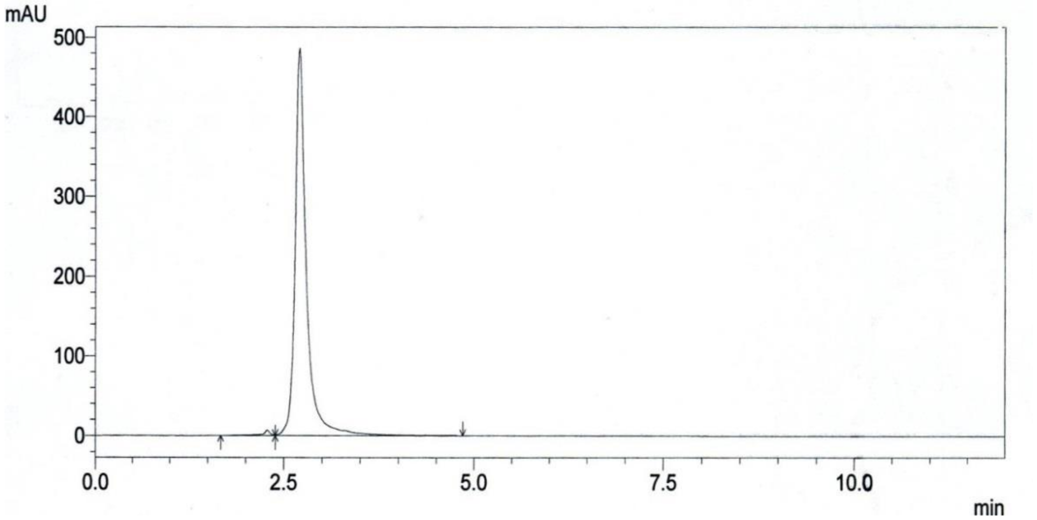

Figure S10. HPLC for compound **11.** The Vydac TP C18; mobile phase CH_3OH; the flow rate 1.0 ml/min; the retention time was 2.68 min; UV detection was carried out at λ 254 nm; the purity was 98.6±0.8%.

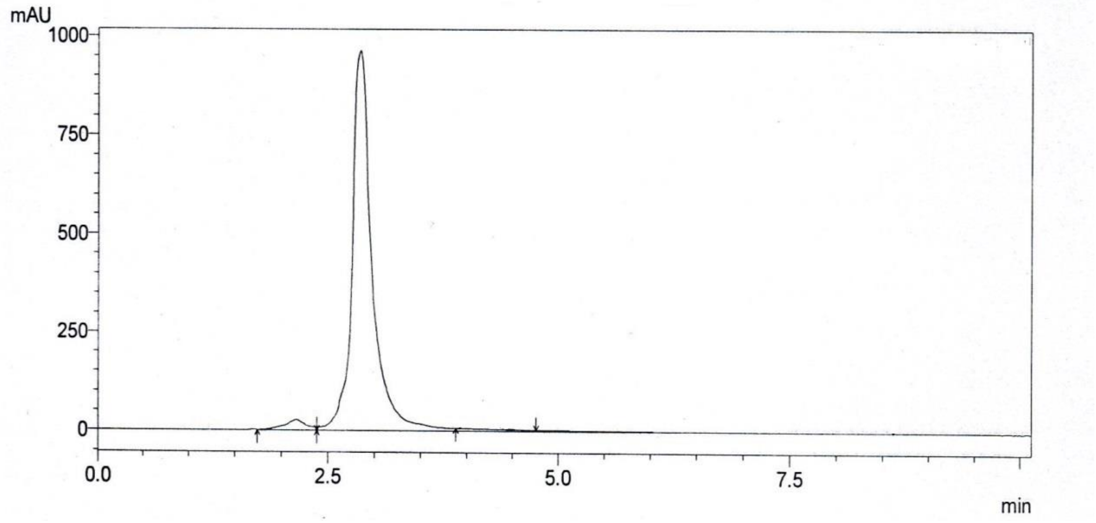

Figure S11. HPLC for compound **12**. A Vydac TP C18 column; a mobile phase CH_3OH; a flow rate 1.0 ml/min; a retention time was 2.84 min; UV detection was carried out at λ 254 nm; the purity was 96.2±0.8%.

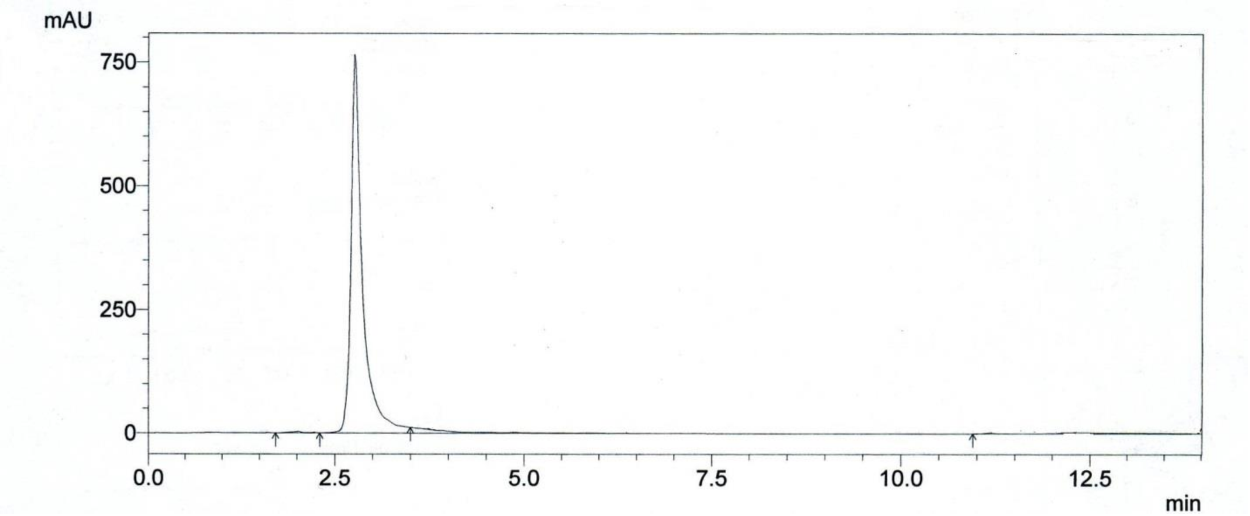

Figure S12. HPLC for compound **13**. The Vydac TP C18 column, a mobile phase CH_3OH; the flow rate 1.0 ml/min; the retention time was 2.76 min; UV detection was carried out at λ 254 nm; the purity was 98.4±0.8%.

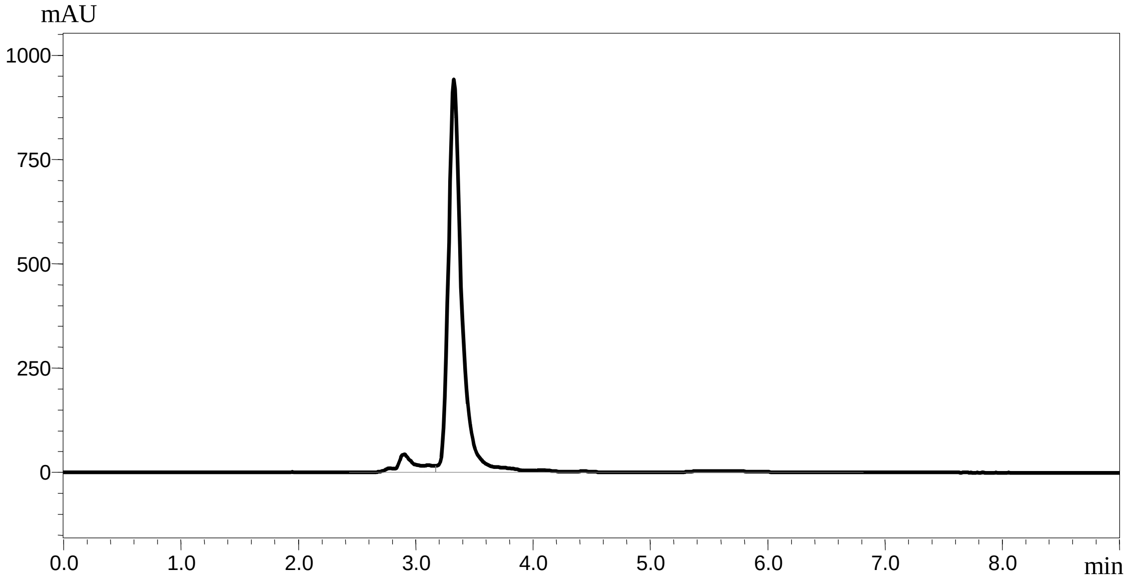

Figure S13. HPLC for compound **14.** The Hypersil ODS C18 column, mobile phase CH_3OH; the flow rate 1.0 ml/min; the retention time was 3.32 min; UV detection was carried out at λ 254 nm; the purity was 95.9±0.8%.

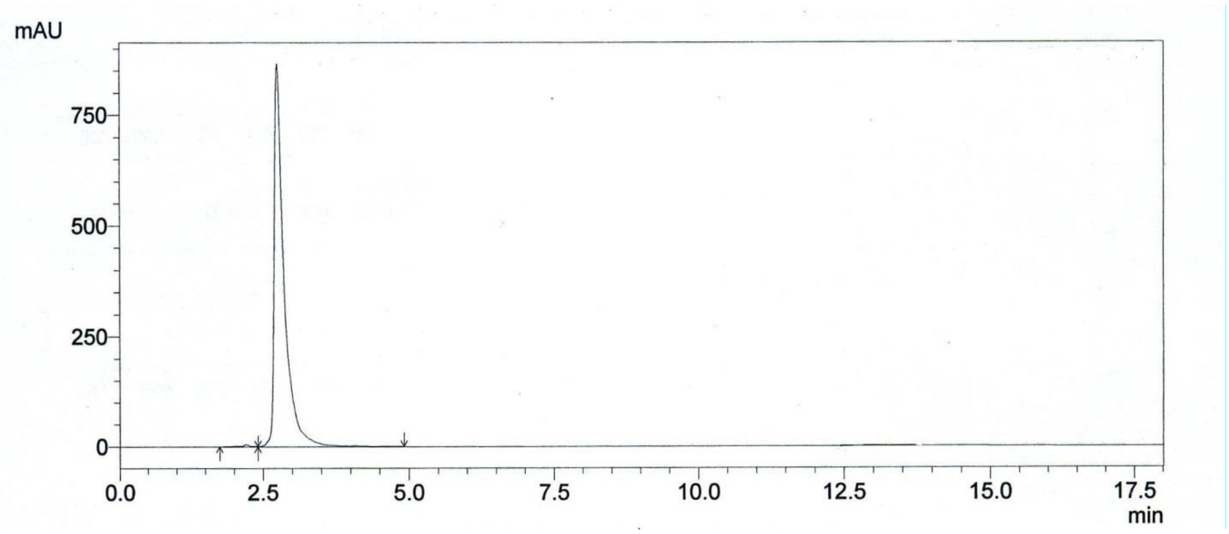

Figure S14. HPLC for compound **15.** The Vydac TP C18, a mobile phase CH_3OH; the flow rate 1.0 ml/min; the retention time was 2.74 min; UV detection was carried out at λ 254 nm; the purity was 98.8±0.8%.

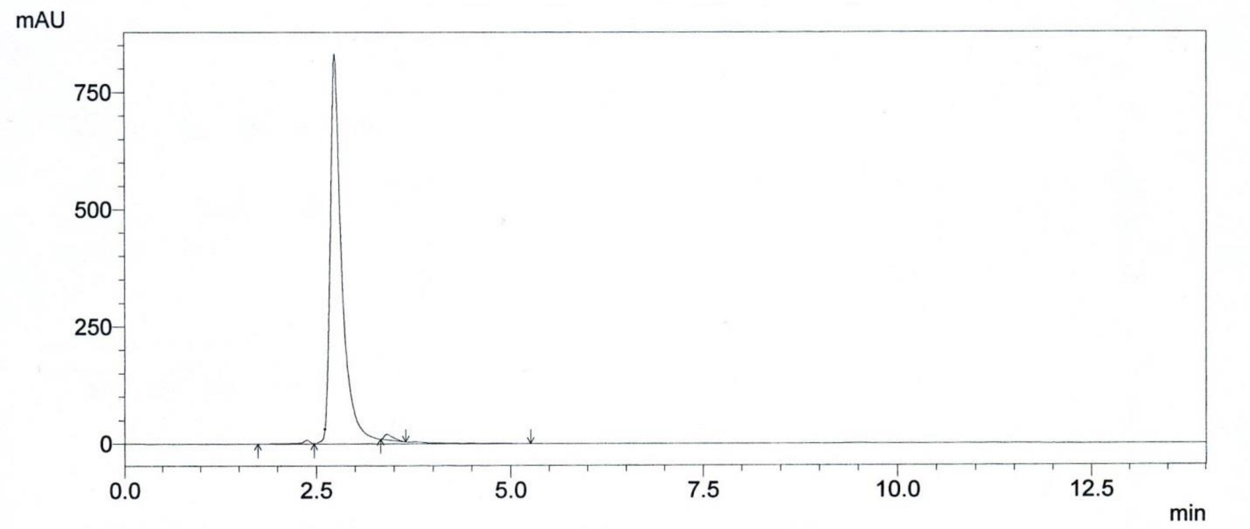

Figure S15. HPLC for compound **16.** The Vydac TP C18 column, mobile phase CH_3OH; the flow rate 1.0 ml/min; the retention time was 2.74 min; UV detection was carried out at λ 254 nm; the purity was 98.2±0.8%.

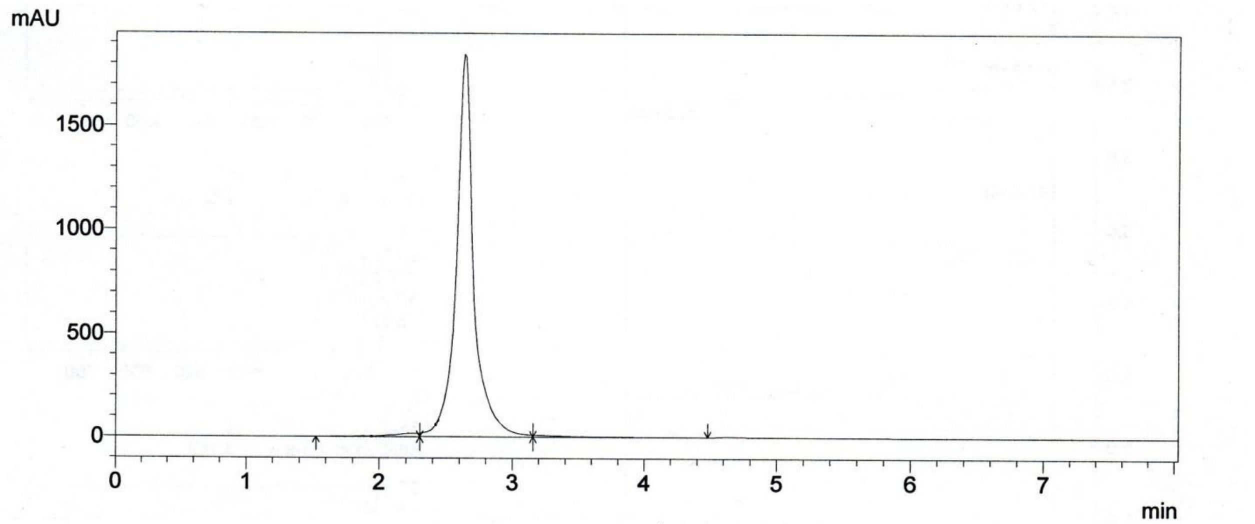

Figure S16. HPLC for compound **17.** The Vydac 218 TPC18 column, USA), mobile phase CH_3OH; the flow rate 1.0 ml/min; the retention time was 2.63 min; UV detection was carried out at λ 254 nm; the purity was 97.5±0.8%.

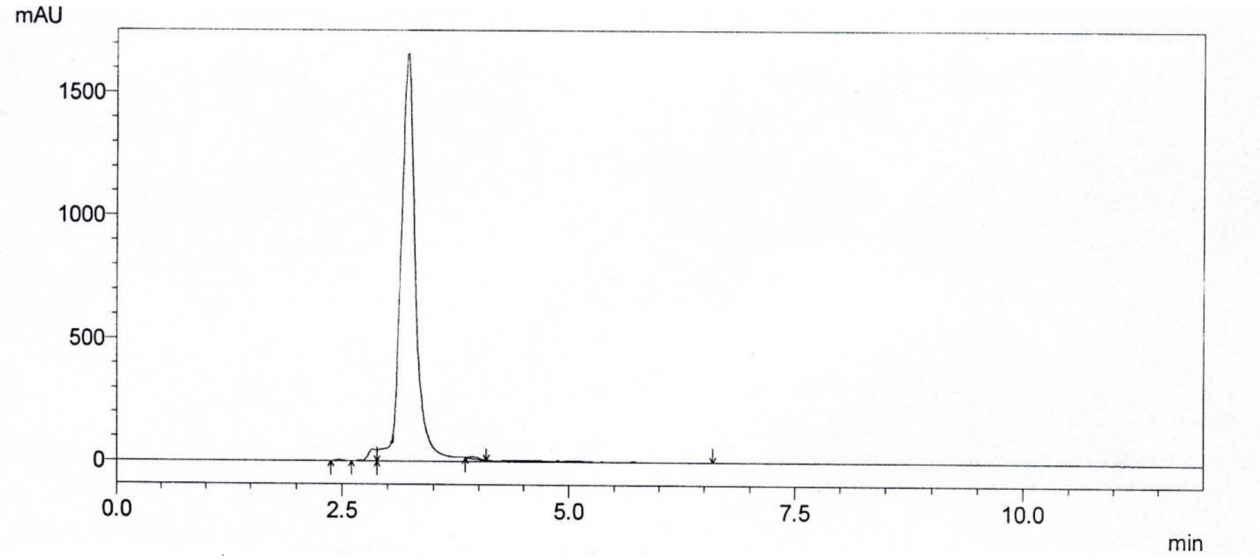

Figure S17. HPLC for compound **18.** The Hypersil ODS C18 column, mobile phase CH_3OH; the flow rate 1.0 ml/min; the retention time was 3.22 min; UV detection was carried out at λ 254 nm; the purity was 97.0±0.8%.

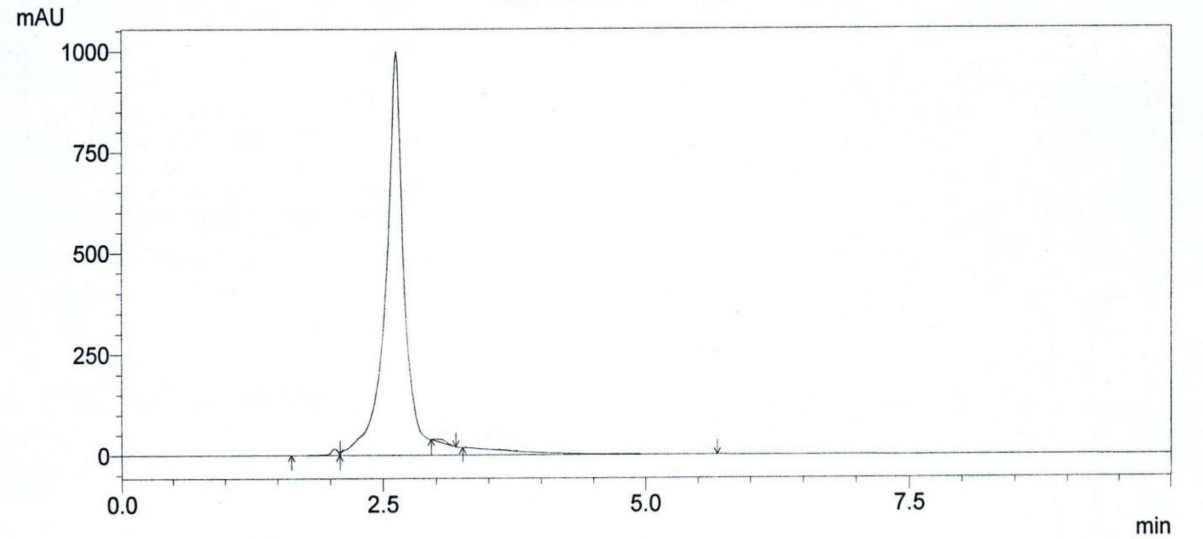

Figure S18. HPLC for compound **19.** The Vydac C18 column, mobile phase CH_3OH; the flow rate 1.0 ml/min; the retention time was 2.62 min; UV detection was carried out at λ 254 nm; the purity was 97.8±0.8%.

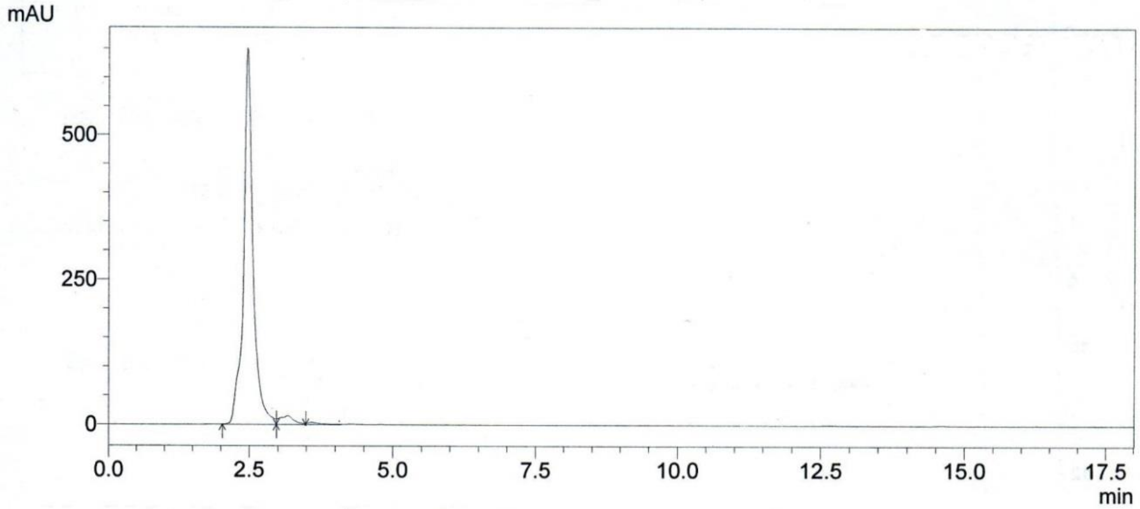

Figure S19. HPLC for compound **20.** The Zorbax C18 column, mobile phase CH_3OH; the flow rate 1.0 ml/min; the retention time was 2.46 min; UV detection was carried out at λ 254 nm; the purity was 96.8±0.8%.

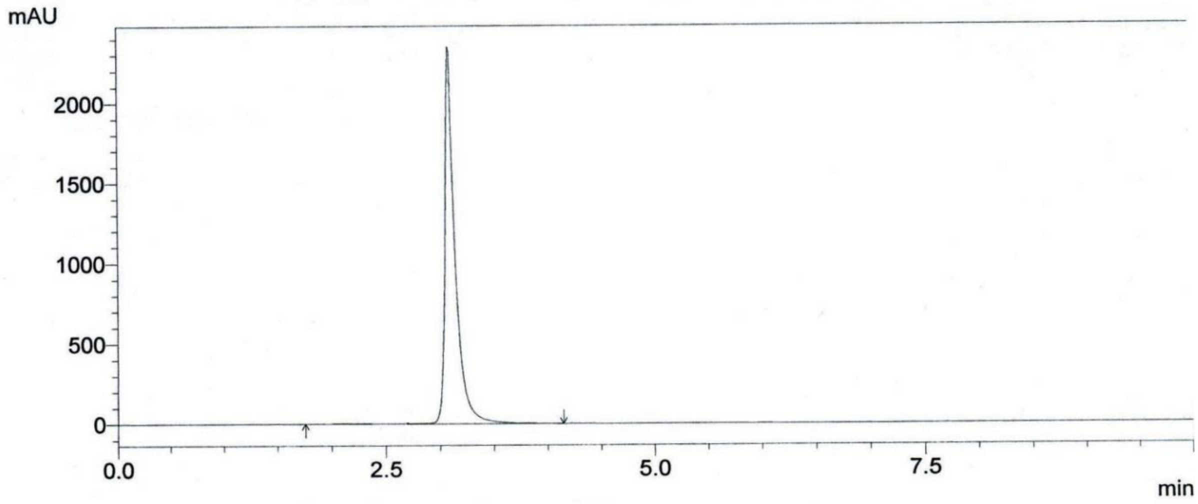

Figure S20. HPLC for BocGly-TyrOMe. The Discovery C18 column; mobile phase CH_3OH; the flow rate 1.0 ml/min; the retention time was 3.08 min; UV detection was carried out at λ 254 nm; the purity was 99.2±0.8%.

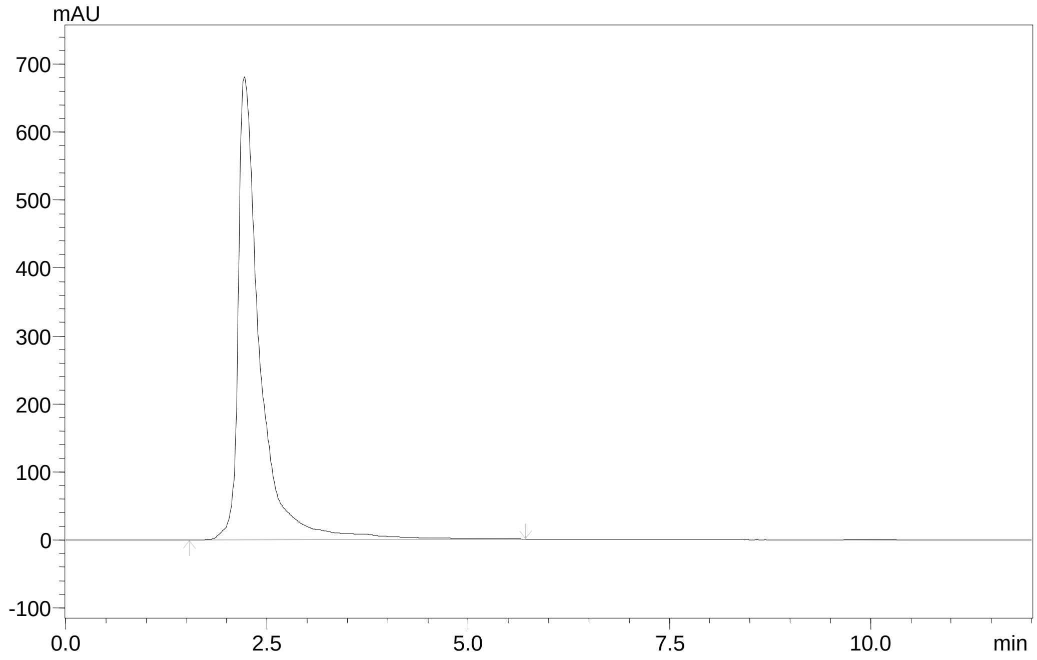

Figure S21. HPLC for compound **21**. The Zorbax RX-C18 column; mobile phase CH_3OH; the flow rate 1.0 ml/min; the retention time was 2.42 min; UV detection was carried out at λ 254 nm; the purity was 98.5±0.8%.

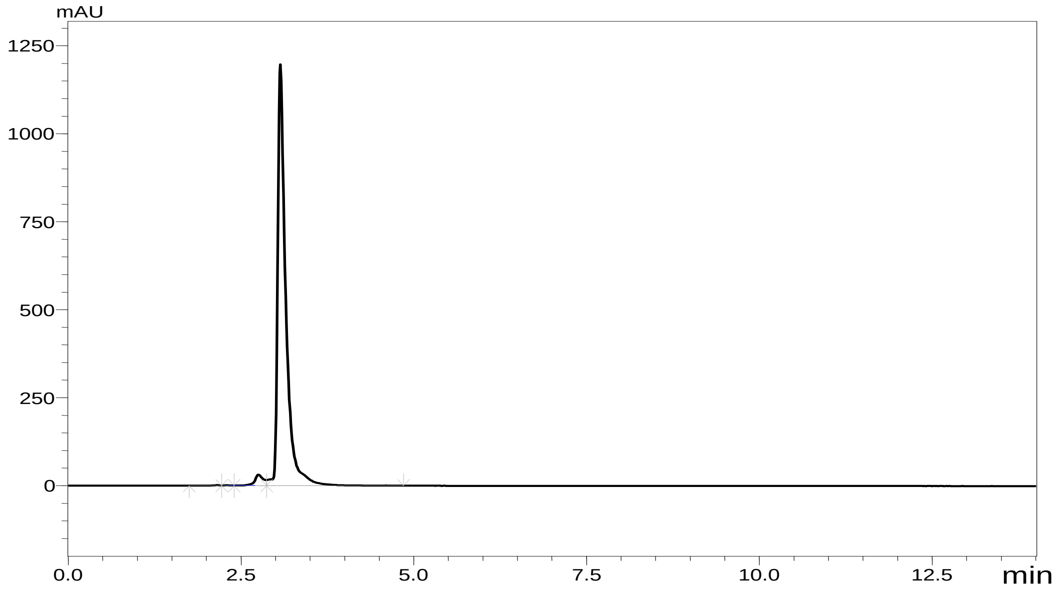

Figure S22. HPLC for dipeptide Boc-Ile-TyrOMe. The Discovery C18 column; the mobile phase CH_3OH; the flow rate 1.0 ml/min; the retention time was 3.10 min; UV detection was carried out at λ 254 nm; the purity was 97.3 ±0.8%.

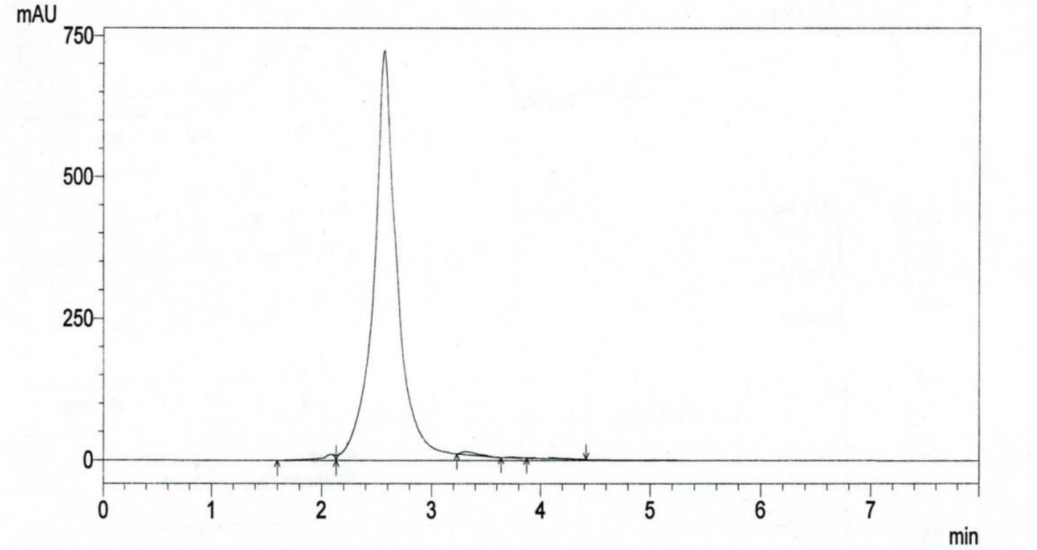

Figure S23. HPLC for compound **22.** The Vydac C18 column; the mobile phase CH_3OH; the flow rate 1.0 ml/min; the retention time was 2.56 min; UV detection was carried out at λ 254 nm; the purity was 96.0 ±0.8%.

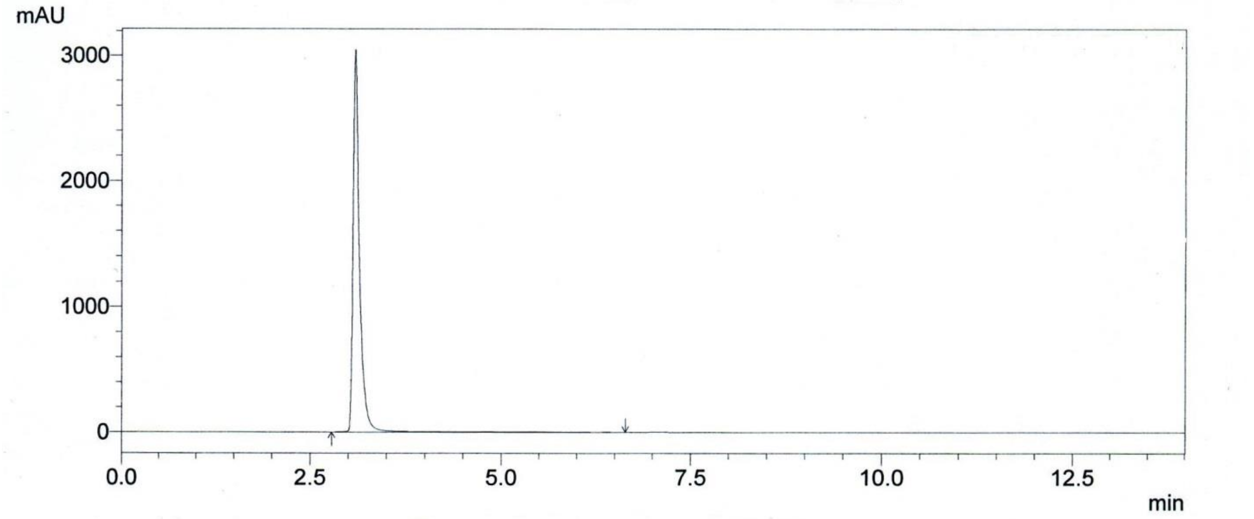

Figure S24. HPLC for dipeptide Boc-Ile-PheOMe**.** The Discovery C18 column; mobile phase CH_3OH; the flow rate 1.0 ml/min; the retention time was 3.08 min; UV detection was carried out at λ 254 nm; the purity was 99.8 ±0.8%.

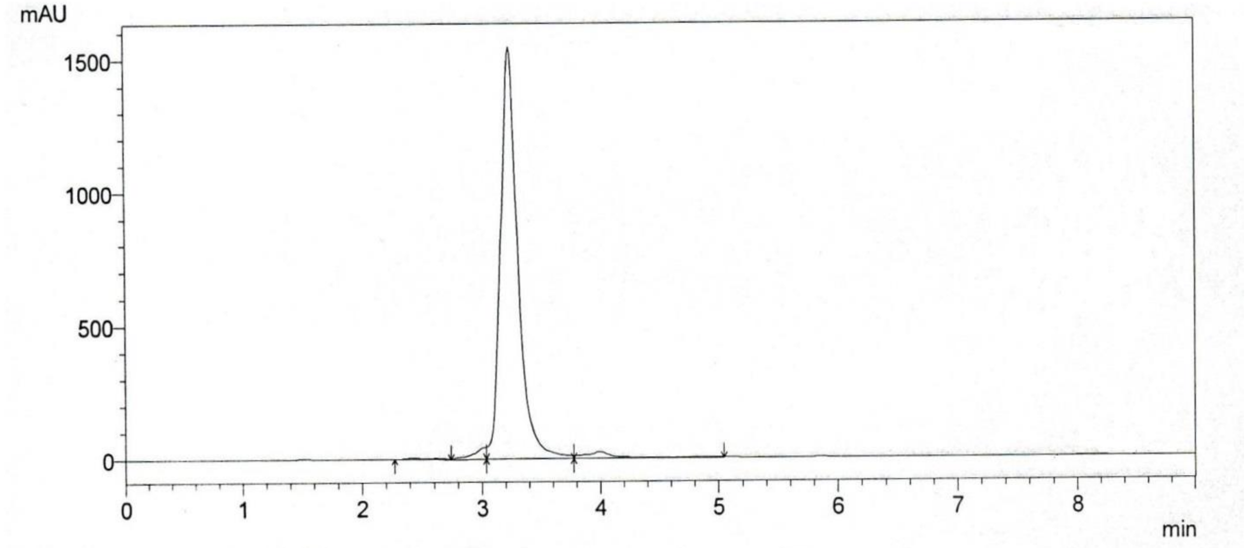

Figure S25. HPLC for compound **23**. The Discovery C18 column; mobile phase CH_3OH; the flow rate 1.0 ml/min; the retention time was 3.24 min; UV detection was carried out at λ 254 nm; the purity was 96.0±0.8%.

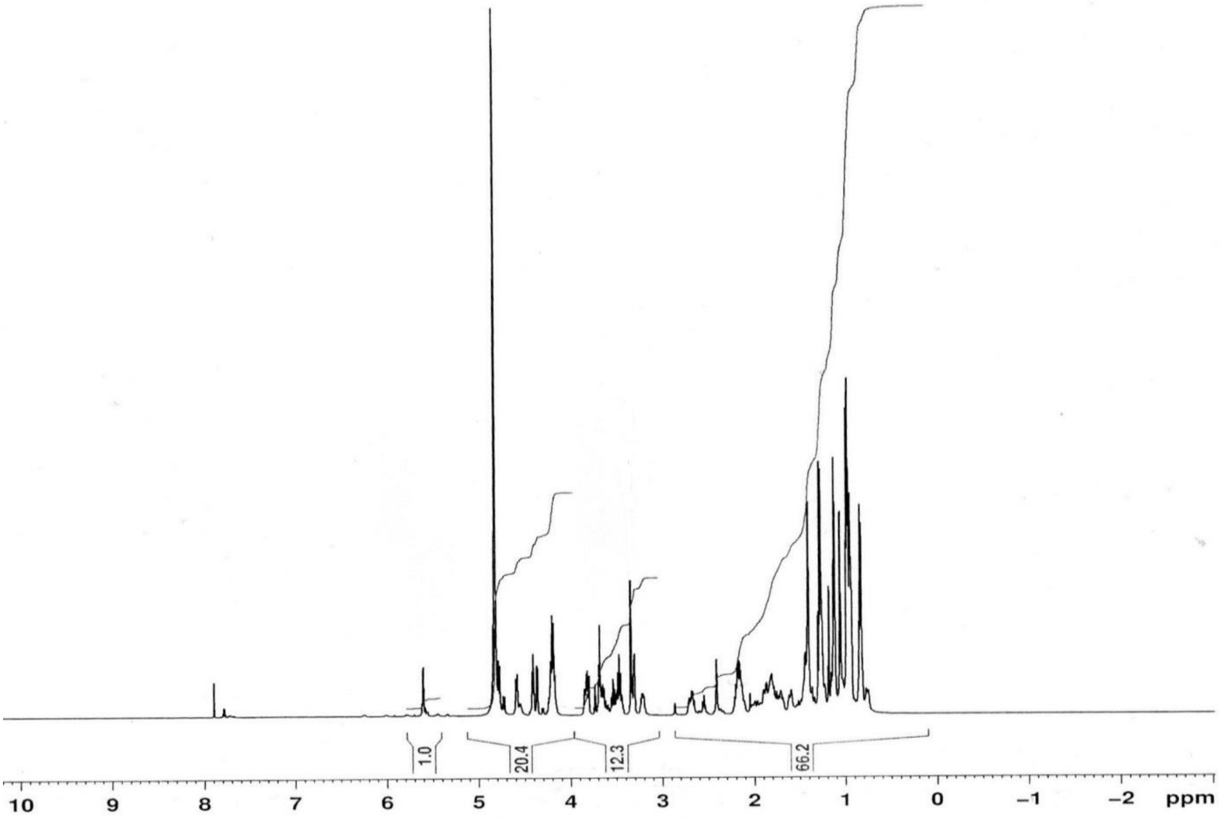

Figure S26. 1H NMR for compound **6** (500 MHz, CD_3OD, δ, ppm)

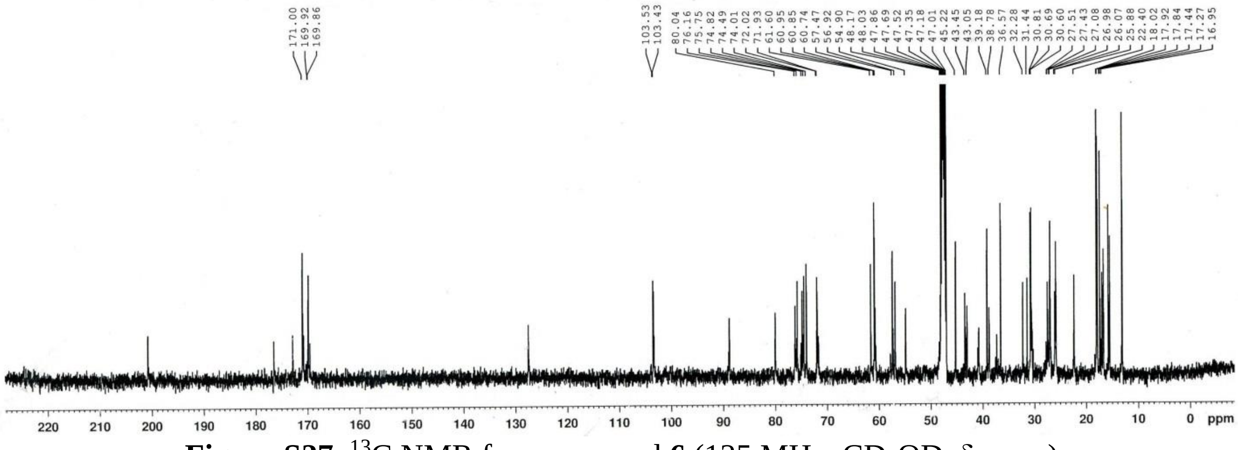

Figure S27. ^{13}C NMR for compound **6** (125 MHz, CD_3OD, δ, ppm)

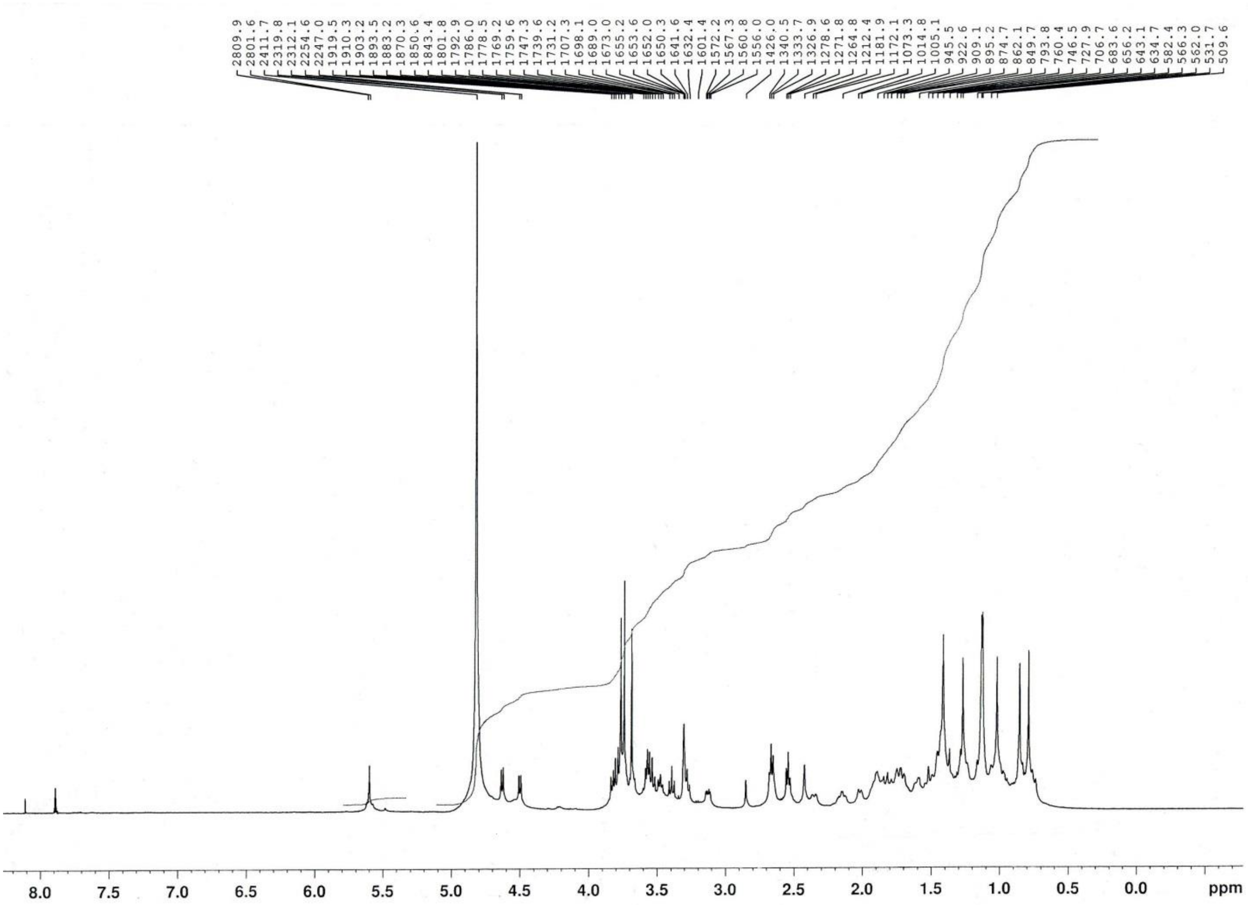

Figure S28. ^{1}H NMR for compound **7** (500 MHz, CD_3OD, δ, ppm)

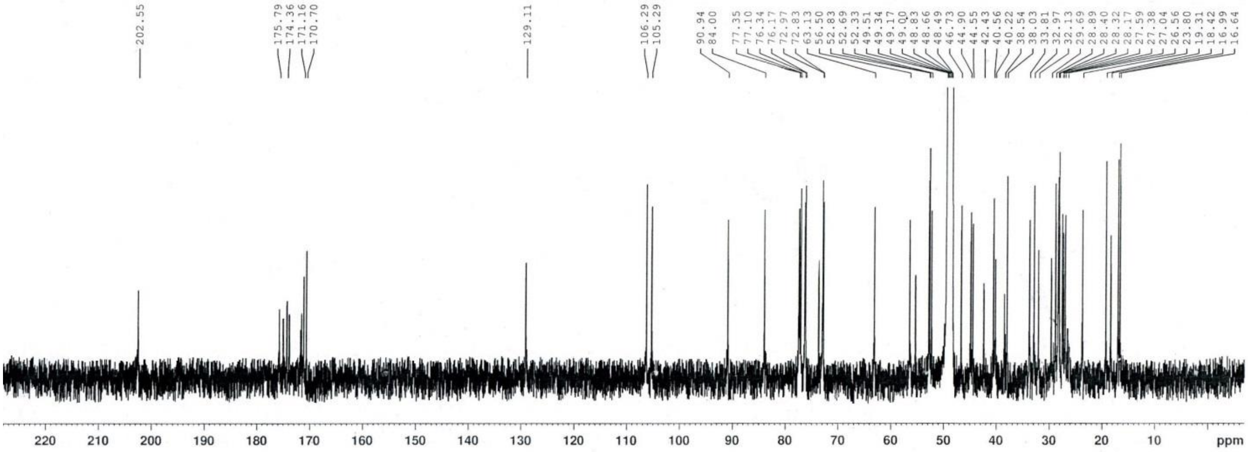

Figure S29. ^{13}C NMR for compound **7** (125 MHz, CD_3OD, δ, ppm)

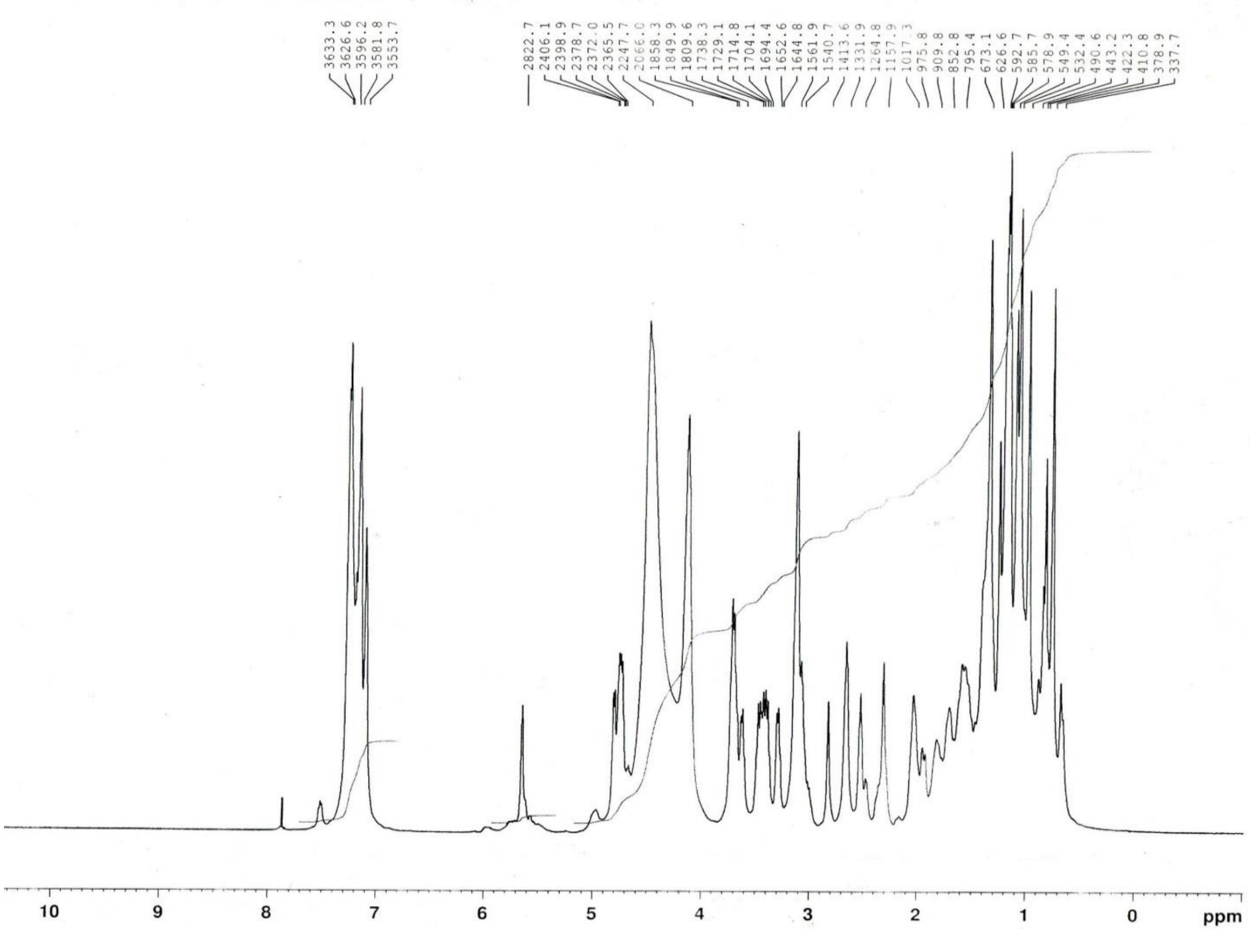

Figure S30. ^{1}H NMR for compound **8** (500 MHz, CD_3OD, δ, ppm)

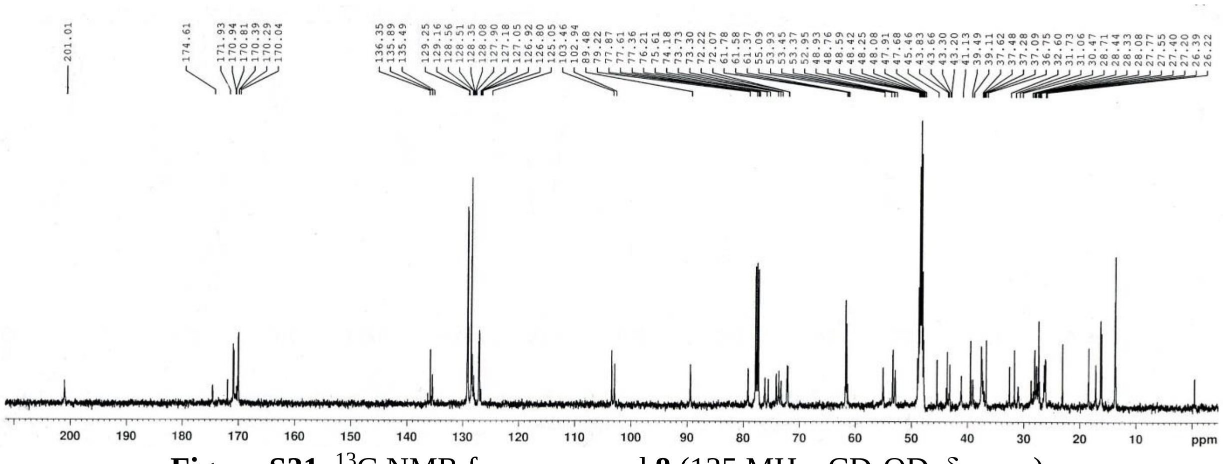

Figure S31. ^{13}C NMR for compound **8** (125 MHz, CD_3OD, δ, ppm)

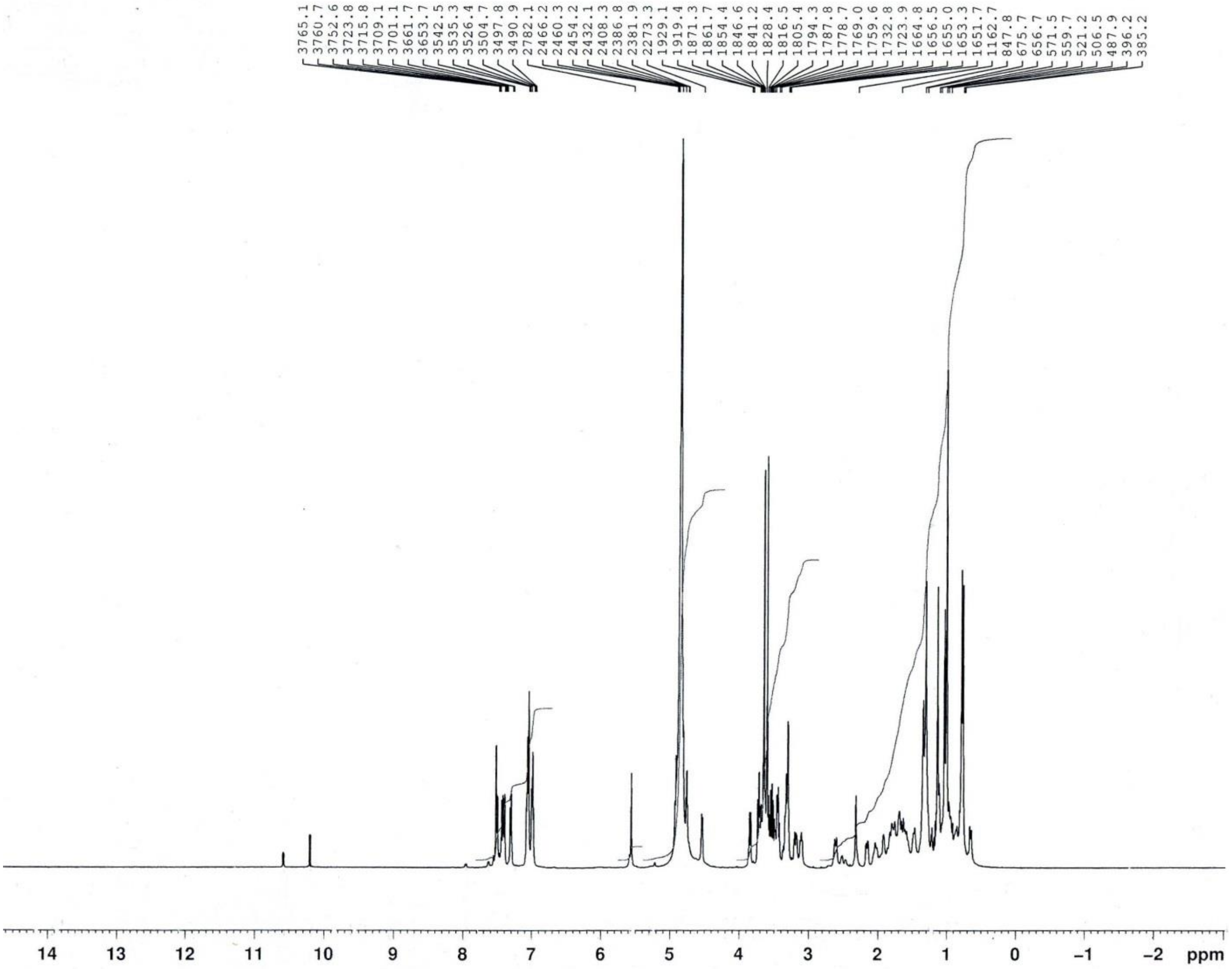

Figure S32. ^{1}H NMR for compound **14** (500 MHz, CD_3OD, δ, ppm)

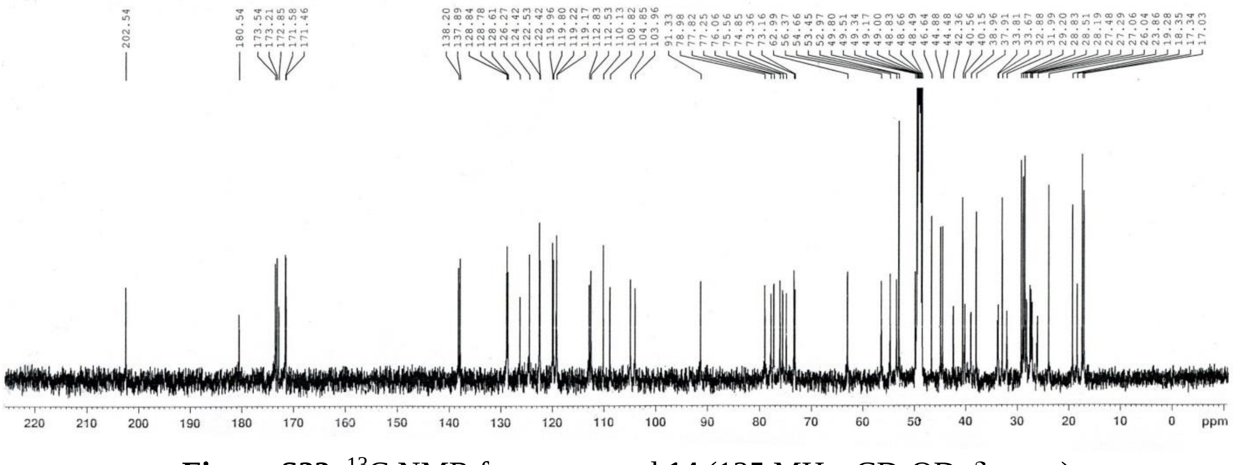

Figure S33. ^{13}C NMR for compound **14** (125 MHz, CD_3OD, δ, ppm)

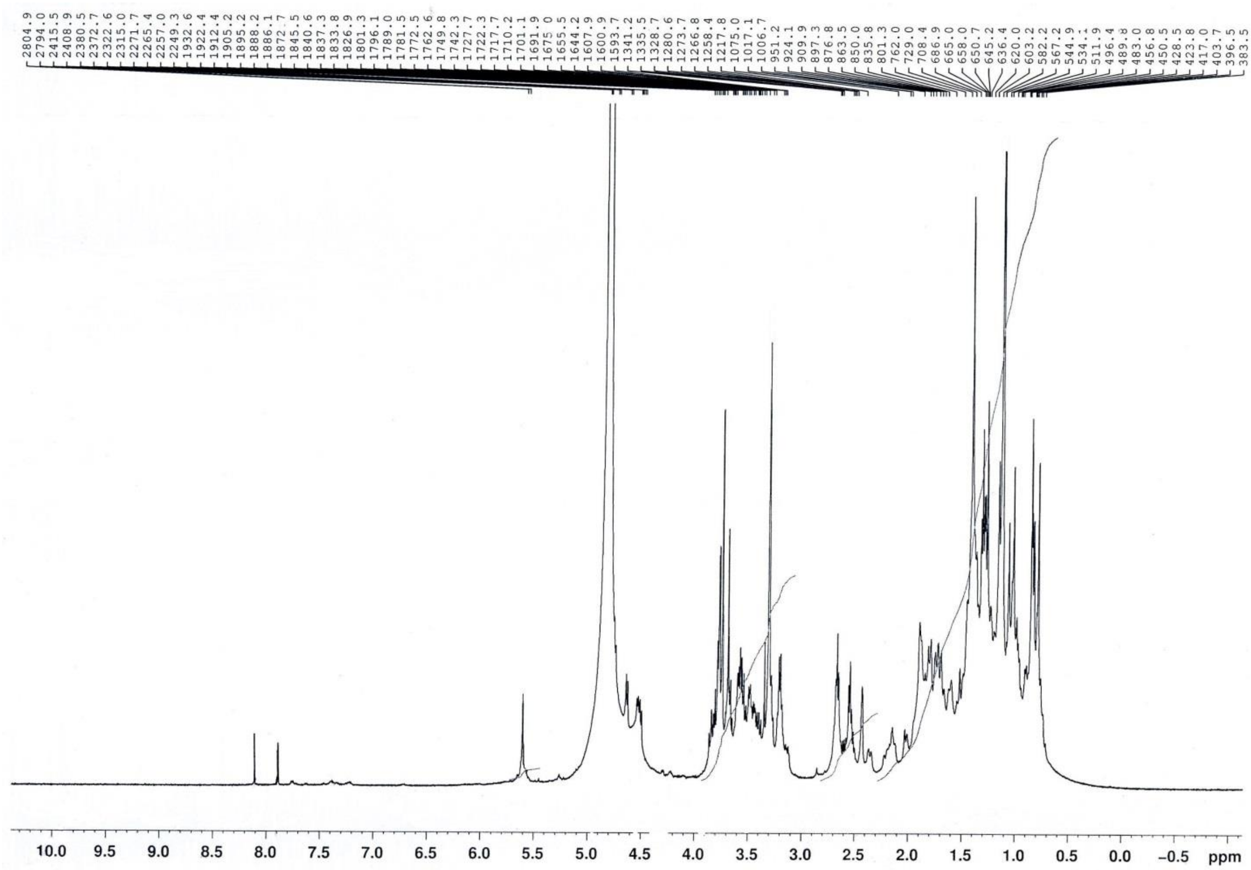

Figure S34. ^{1}H NMR for compound **15** (500 MHz, CD_3OD, δ, ppm)

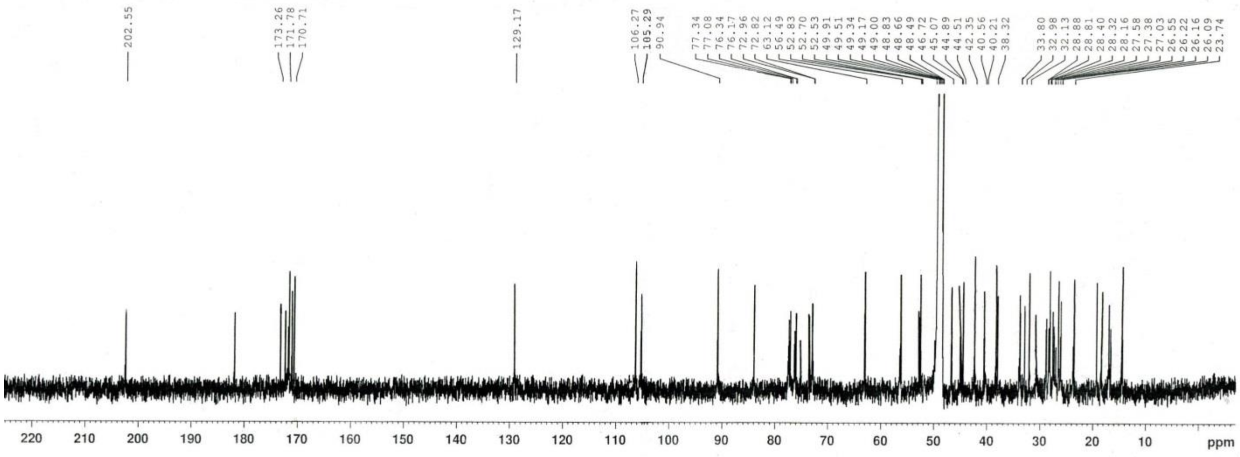

Figure S35. ^{13}C NMR for compound **15** (125 MHz, CD_3OD, δ, ppm)

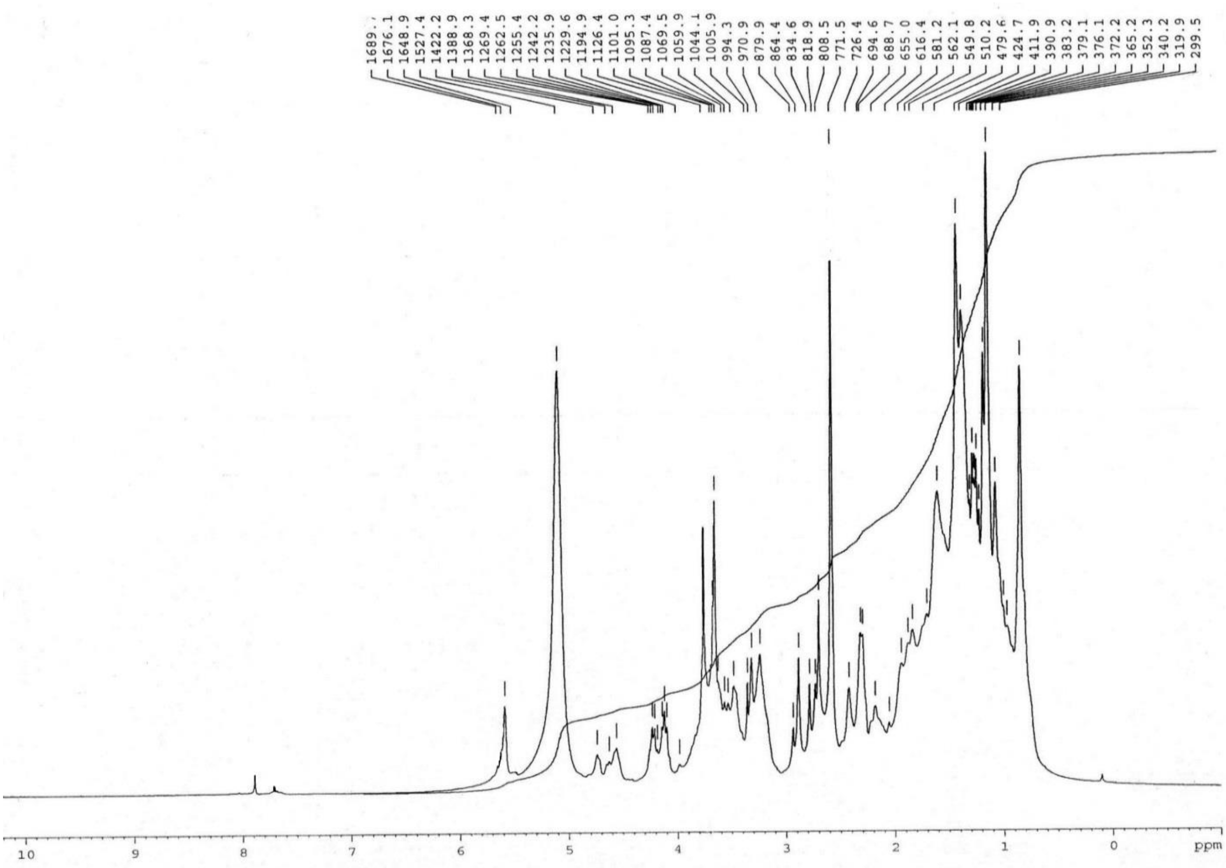

Figure S36. ^{1}H NMR for compound **19** (500 MHz, D_3OD, δ, ppm)

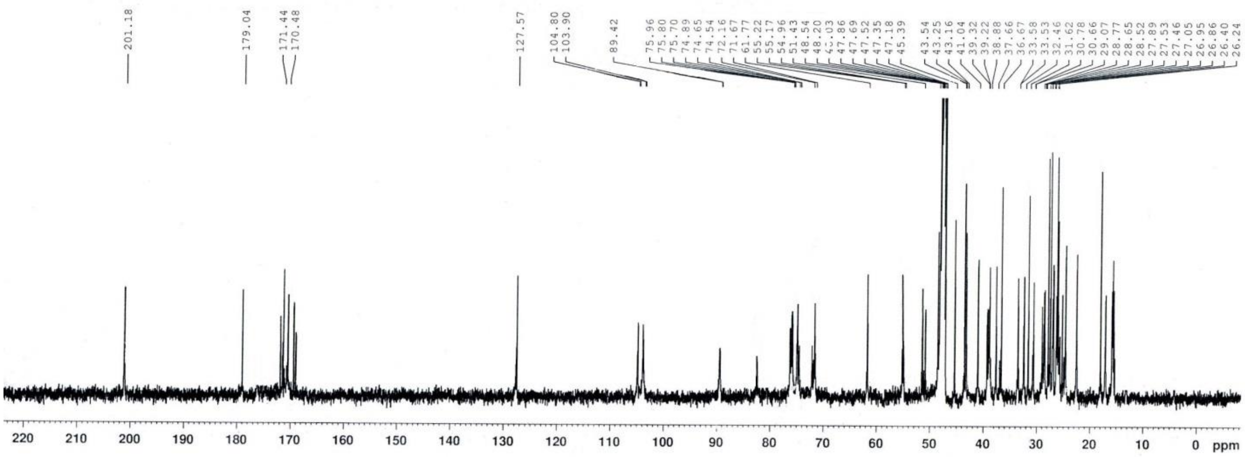

Figure S37. ^{13}C NMR for compound **19** (125 MHz, CD_3OD, δ, ppm)

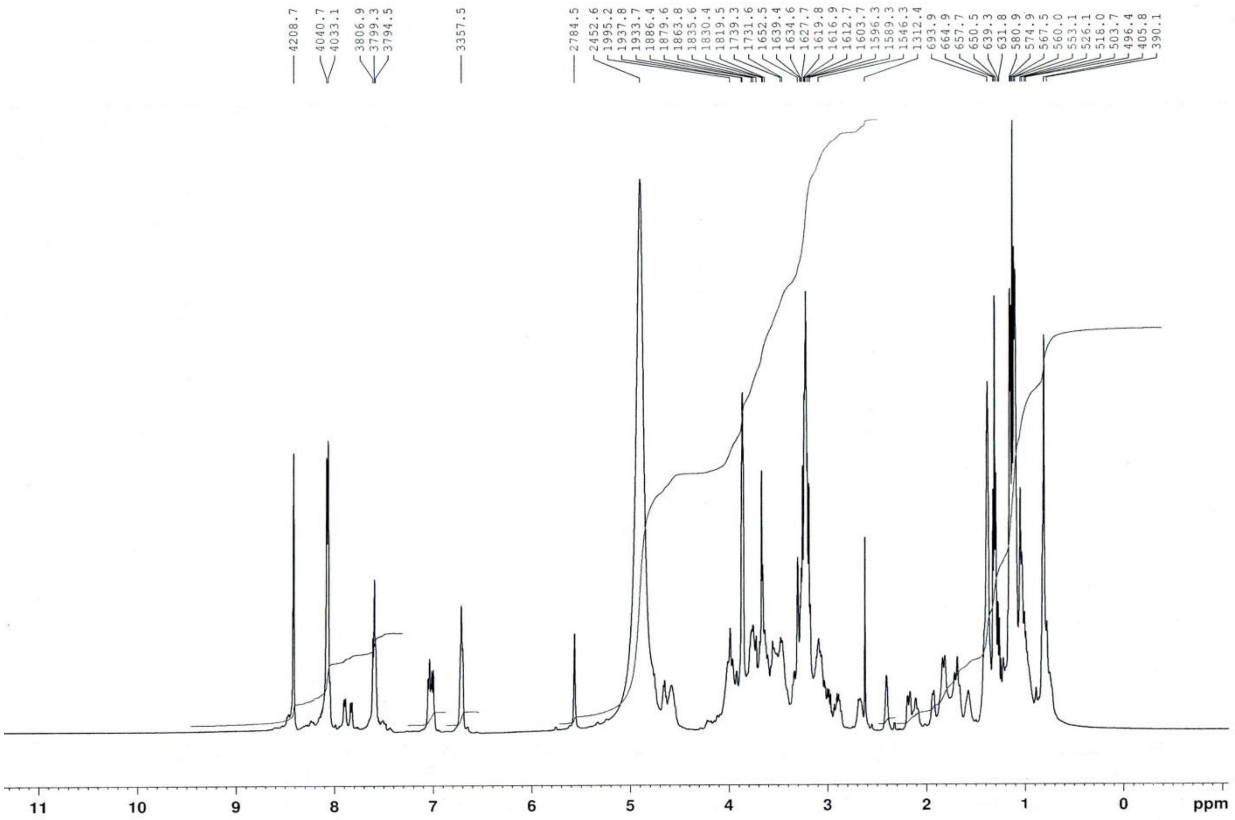

Figure S38. ^{1}H NMR for compound **21** (500 MHz, CD_3OD, δ, ppm)

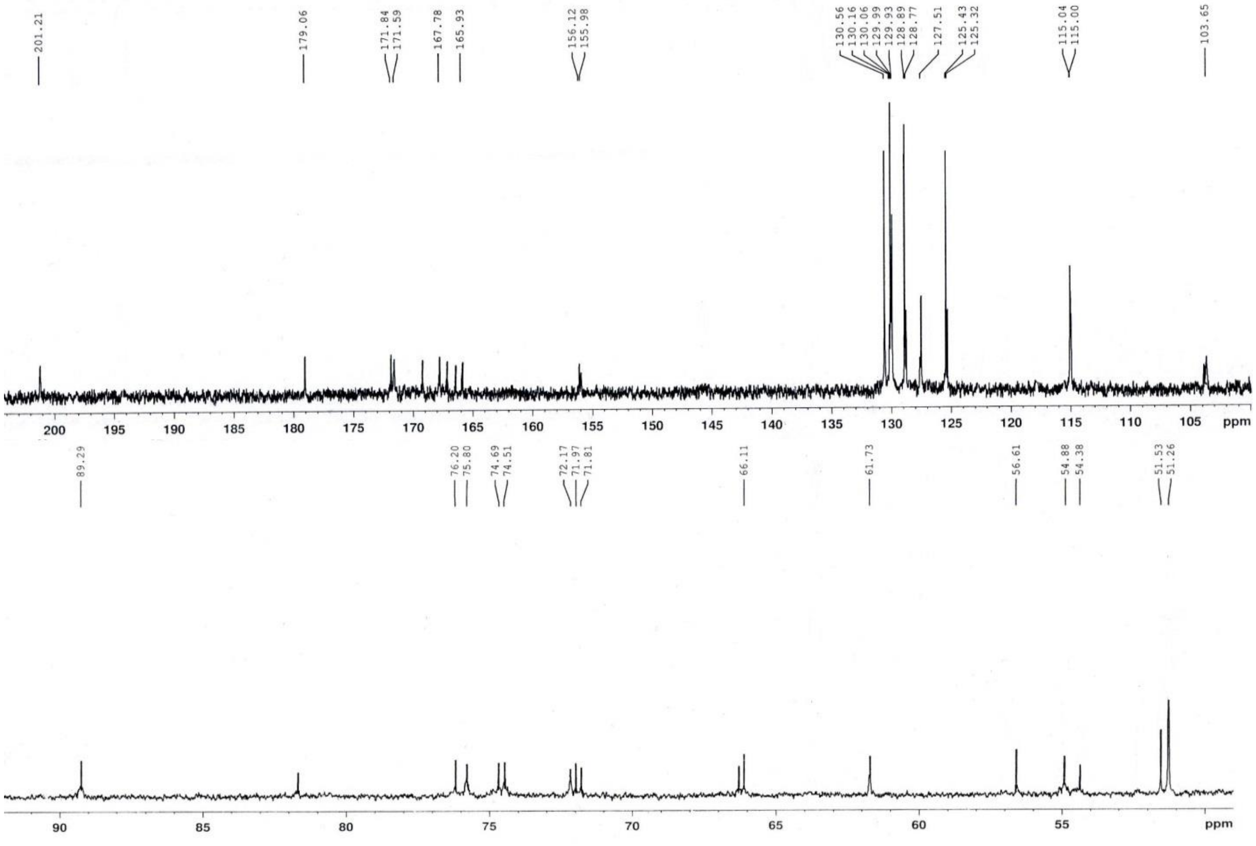

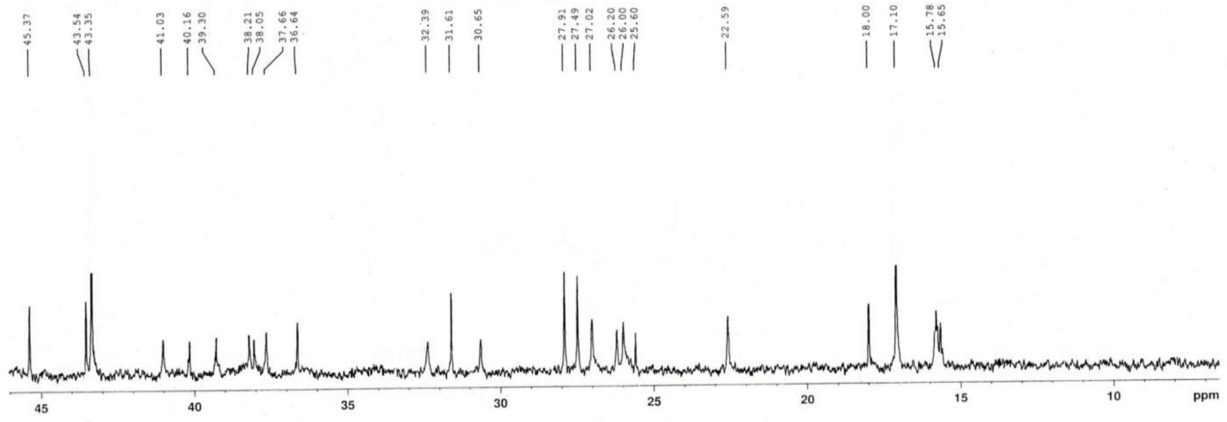

Figure S39. ^{13}C NMR for compound **21** (125 MHz, CD_3OD, δ, ppm)

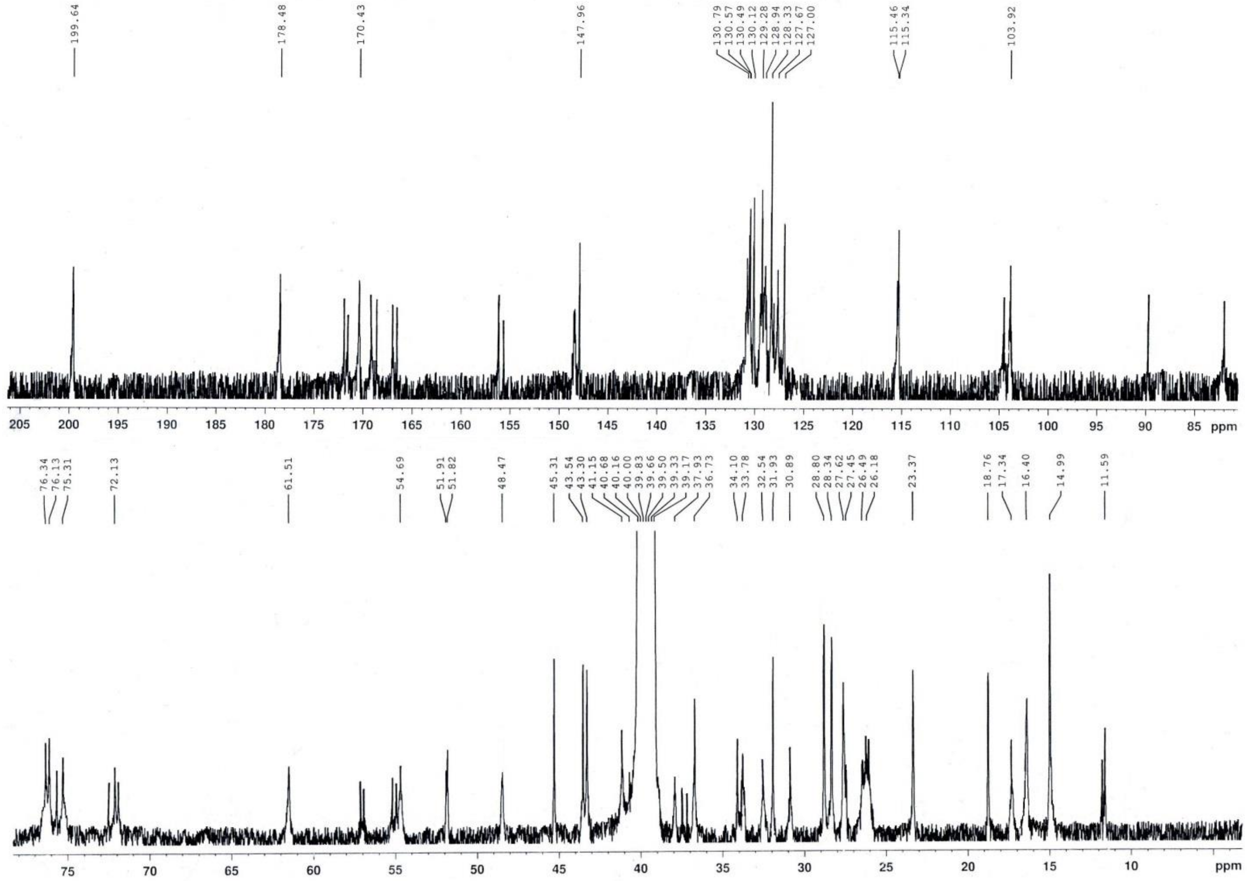

Figure S40. ^{13}C NMR for compound **22** (125 MHz, DMSO-d_6, δ, ppm)

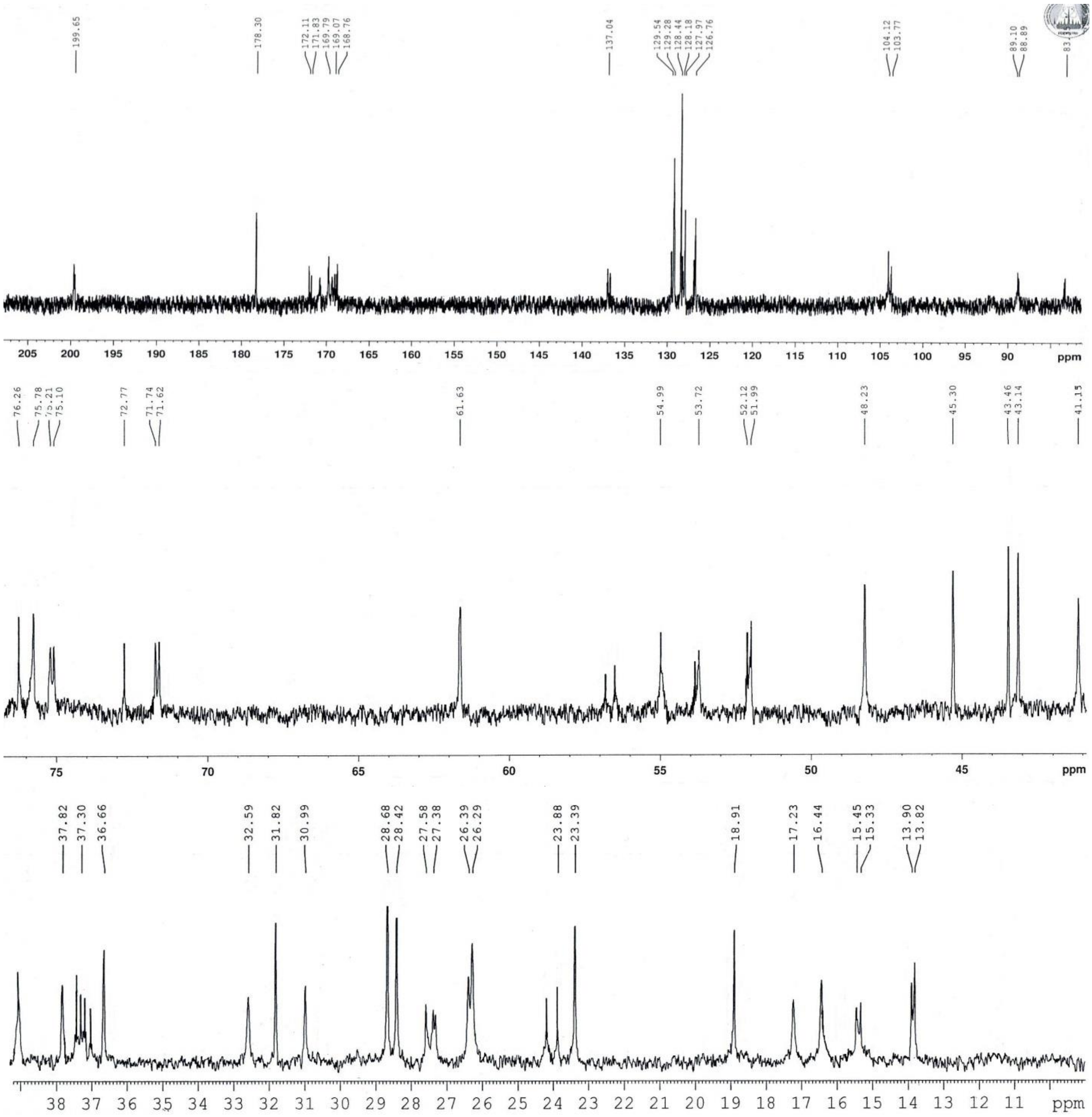

Figure S41. ^{13}C NMR for compound **23** (125 MHz, DMSO-d_6, δ, ppm)

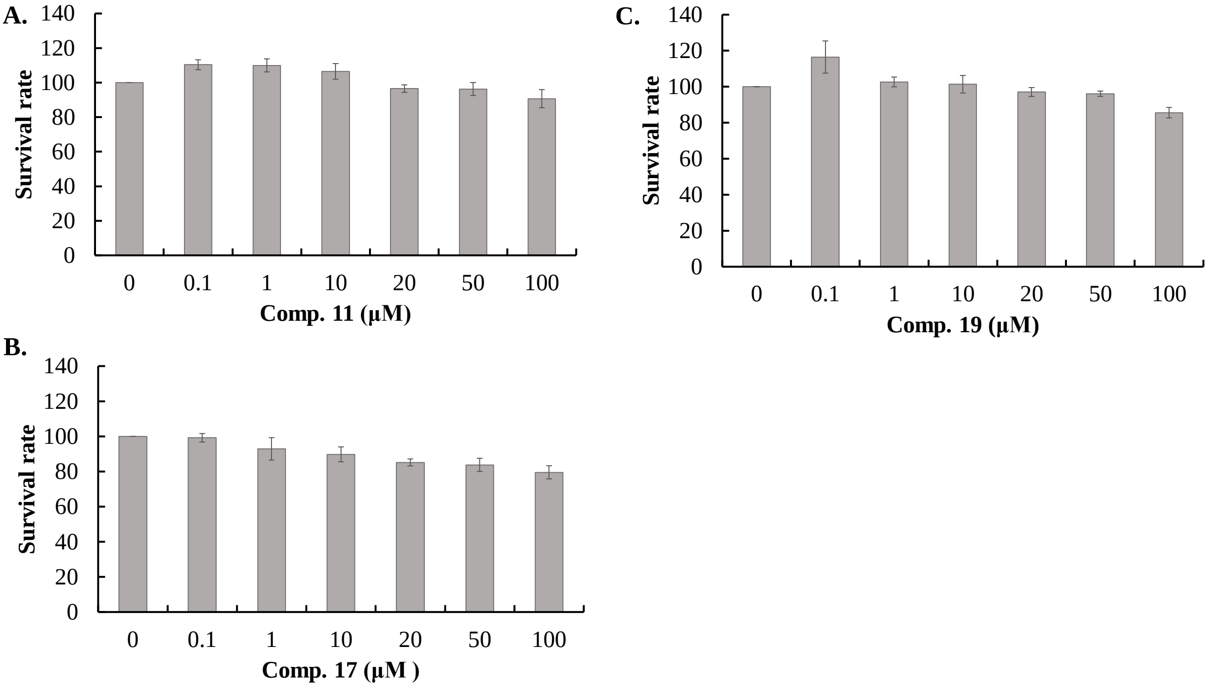

Figure S42. The cytotoxicity of active compounds was assessed on Vero E6 cells using the MTT assay. Cells were treated with varying concentrations of compounds 11 (A), 17 (B), and 19 (C) for 96 hours. Following treatment, MTT solution was added, and the cells were incubated for an additional 4 hours at 37 °C. Cell viability (%) was then determined by measuring MTT reduction.
